# Prioritizing Detailed Item Memories Through Post-Encoding Motivation Requires Consolidation

**DOI:** 10.1162/OPMI.a.379

**Published:** 2026-07-17

**Authors:** Taylor A. Chamberlain, Daphna Shohamy, Christopher Baldassano

**Affiliations:** Department of Psychology, Columbia University, New York, NY, USA; Zuckerman Mind Brain Behavior Institute, Columbia University, New York, NY, USA; Kavli Institute for Brain Science, Columbia University, New York, NY, USA

**Keywords:** episodic memory, motivation, events, reward, consolidation

## Abstract

Prioritizing memories that are motivationally salient is adaptive for goal-directed behavior, ensuring that information being retained is the most relevant to future goals. Past research demonstrates we can use value signals to adaptively modulate our memory. Crucially, however, the timing of value signals varies. Sometimes we know the significance of an upcoming event in advance, while other times, we are unaware of an event’s importance until after the fact. Does the timing of a value signal relative to encoding change the cognitive mechanism by which it shapes memory? To test this question, we developed a motivated encoding task where the motivational cue appeared either before or after encoding. Across two independent samples, pre-encoding motivation improved item memory regardless of test timing, while the benefit of post-encoding motivation only appeared after a 24-hour delay. For source memory, pre-encoding motivation reliably enhanced performance across conditions. In contrast, the effect of post-encoding motivation on source memory was more variable: it was significant across both test timings in Experiment 2, but was weaker and more variable in Experiment 1. Furthermore, the two conditions differently affected incidental memory organization; pre-encoding motivation enhanced item order memory relative to post-encoding motivation. These results reveal the dissociable effects of pre- and post-encoding motivation, and suggest that when we learn of an event’s importance might determine the mechanism by which we can strengthen its memory for future use.

## INTRODUCTION

The ability to prioritize what we remember is crucial for ensuring that our memories align with our goals. Research on the effects of motivation on memory suggests that cues about the importance of information, such as its potential monetary value, can enhance memory (Adcock et al., 2006; Gholston et al., 2023; Gruber et al., 2013; Murty et al., 2011; Shigemune et al., 2010, 2014, 2017; Spaniol et al., 2014; Villaseñor et al., 2021; Wolosin et al., 2012). A common task for studying this process is the monetary incentive encoding task (Adcock et al., 2006), where value cues presented during encoding denote the amount of reward a participant will receive if they correctly remember a given item on a subsequent memory test. Past work demonstrates that motivation enhances recognition memory (Adcock et al., 2006; Gholston et al., 2023; Gruber et al., 2013; Shigemune et al., 2010; Spaniol et al., 2014; Villaseñor et al., 2021), associative memory (Shigemune et al., 2017; Wolosin et al., 2012), and, to some extent, source or context memory (Gholston et al., 2023; Shigemune et al., 2014; Villaseñor et al., 2021) although see: (Gholston et al., 2023; Shigemune et al., 2014; Villaseñor et al., 2021). However, despite substantial progress in characterizing motivation’s enhancement on different kinds of memory, several questions about the cognitive mechanisms governing this enhancement remain open.

One key unresolved question is how the timing of motivation impacts the ability to prioritize memory. While experiments typically present motivational value cues pre-encoding, in daily life we don’t always know which information will be important in advance. A teacher might prompt students to remember an upcoming slide, but could just as well underscore its importance only after the fact, remarking, “That last slide will be on the exam.” Receiving a value signal at the end of an episode has also been a central focus of reinforcement learning models, which predict that this feedback drives updates to your internal models of the world (Gershman & Daw, 2017). When the value of a stimulus is revealed after encoding, rather than before encoding, does it influence memory through the same mechanisms?

Prior work proposes that motivation initiates anticipatory midbrain processes, which, via dopaminergic modulation, strengthen memory formation in the medial temporal lobe (Adcock et al., 2006; Cohen et al., 2017; Knowlton & Castel, 2022; Poh et al., 2022; Shohamy & Adcock, 2010). Memory enhancement has also been shown to benefit from selective attention (Cohen et al., 2017; Elliott & Brewer, 2019; Knowlton & Castel, 2022; Murphy et al., 2024; Yin et al., 2024) and strategic encoding (Cohen et al., 2014). However, such mechanisms are only relevant for memory that is preceded by a motivationally relevant cue, suggesting they would not be effective routes to memory enhancement due to post-encoding motivation. Instead, memories can be selectively strengthened post-encoding through offline processes that happen following the event.

Research on incidental reward has shown evidence for this kind of after-the-fact prioritization; receiving a reward enhances memory for neutral items encoded prior to the reward (Braun et al., 2018; Dunsmoor et al., 2022; Knowlton & Castel, 2022; Lin et al., 2025; Murayama & Kitagami, 2014; Nielson & Bryant, 2005; Patil et al., 2016). Notably, these effects appear only after a consolidation period (Cowan et al., 2021), with some evidence suggesting a graded pattern, in which enhancement is stronger for stimuli closer to the reward. These effects might arise through increased rates of reverse hippocampal replay in response to reward, as has been shown in rodents (Ambrose et al., 2016; Shohamy & Adcock, 2010). Human fMRI research corroborates this theory; rewarded items are preferentially reactivated during sleep and post-task rest (Gruber et al., 2016; Sterpenich et al., 2021) and post-encoding connectivity between the VTA, hippocampus, and category-selective visual cortex selectively predicts memory for high-reward memoranda (Murty et al., 2017). This line of work raises the possibility that motivational cues, much like direct receipt of reward, could trigger similar consolidation-based processes which prevent important memories from decaying. Paradigms that manipulate test expectations support this idea: when participants are told after encoding that studied items (or a category of studied items) will be tested later, their performance improves relative to baseline, but only if the test takes place after a night of sleep (van Dongen et al., 2012; Wilhelm et al., 2011). More broadly, consolidation-based mechanisms have been implicated in motivation reward-driven memory enhancement, including when cues are presented before or concurrent with encoding (Adcock et al., 2006; Igloi et al., 2015; Murayama & Kuhbandner, 2011; Wittmann et al., 2005; though reward-driven memory effects have also been observed without a consolidation delay, see: Gholston et al., 2023; Gruber et al., 2013; Murty & Adcock, 2014). Critically, while pre-encoding motivation can enhance memory through both encoding-phase mechanisms and post-encoding consolidation, post-encoding motivation can by definition only operate through mechanisms that take place after the event. This asymmetry leads to the prediction that the memory benefit of post-encoding motivation should be uniquely dependent on consolidation.

An alternative possibility is that motivation enhances memory through similar mechanisms, regardless of its timing relative to encoding. Research on event segmentation, the process of dividing continuous experience into discrete episodes, suggests that some memory processes might not take place until after an event ends and could therefore be impacted by either pre- or post-event motivation. Specifically, brain dynamics just after an event are predictive of later recall (Baldassano et al., 2017; Barnett et al., 2022; Ben-Yakov & Dudai, 2011; Ben-Yakov & Henson, 2018), and computational work suggests that boundaries are optimal moments for transferring information from working memory into episodic memory (Lu et al., 2022). Applying this framework to motivated encoding, a value cue presented immediately after an event (i.e., at the boundary) could have the same effect as a cue presented before encoding, as long as the event’s contents are still active in working memory. This perspective would predict that pre- and post-encoding motivation could enhance memory through the same underlying mechanisms.

Therefore there are two alternative hypotheses: (1) Pre- and post-encoding motivation influence memory through distinct mechanisms, such that post-encoding motivation is uniquely dependent on consolidation, or (2) Pre- and post-encoding motivation rely on the same mechanism and are equally affected by the consolidation process. In the present study, we aimed to adjudicate between these hypotheses, by examining motivation timing and consolidation in tandem, and assessing how these factors jointly influence memory. Participants viewed a sequence of images, grouped into four-item “events.” During the study phase, motivation cues indicated whether an event was motivated or neutral, appearing either before the event (*pre-encoding condition*) or after the event (*post-encoding condition*).

While motivation reliably enhances item memory (Adcock et al., 2006; Gruber et al., 2013; Shigemune et al., 2010; Spaniol et al., 2014), its effects on source and associative memory are mixed (Gholston et al., 2023; Shigemune et al., 2014; Villaseñor et al., 2021; Wolosin et al., 2012). To clarify how motivation might differentially impact distinct forms of episodic memory, our paradigm included three types of memory tests. First, during encoding, participants completed an unrewarded temporal order task, designed to assess incidental effects of motivation on memory organization. Second, participants completed an item memory test, where they distinguished between items shown during encoding and similar lures, and received a reward for every motivated item they correctly identified. Lastly, to measure the impact of motivation on source memory, participants identified the color of the border paired with a given image, again receiving a reward for every correct motivated trial. Critically, to test the role of consolidation, the item and source memory tests occurred either 3 minutes or 24 hours after encoding.

Across two independent samples, we find that both pre-encoding (*prospective*) and post-encoding (*retrospective*) motivational cues enhance memory for items and source information. Interestingly, retrospective motivation consistently improved both item and source memory only in the 24-hour delay condition. Conversely, prospective motivation benefited item and source memory in both immediate and 24-hour delay conditions across both experiments. Furthermore, echoing prior work on the effect of reward, we observe a stronger motivation effect for items closer to the cue, but only following a 24-hour delay. Finally, the motivation conditions demonstrated opposing patterns on memory organization: pre-encoding motivation significantly benefited temporal order judgements relative to post-encoding motivation.These findings highlight the role of timing in determining motivation’s impact and suggest multiple pathways through which valuable information can be prioritized in memory.

## METHODS

### Experiment 1

#### Participants.

A total of 596 participants were recruited for Experiment 1 online via the Prolific platform. All individuals included were located in the U.S., proficient in English, possessed normal or corrected vision, and had normal color vision. Of these participants, 26 did not return for the second part of the study, 22 failed attention checks, 35 responded to too few trials (defined as <85% on any given portion of the study), 78 failed one or more instruction comprehension questions, 14 experienced technical errors with the task, and 1 did not complete the task in the allotted time. Thus the final sample consisted of 420 participants (260 female; mean age = 24.98 ± 2.8 years, age range = 18–30 years). Participants breakdown by experimental condition was as follows: prospective-immediate (*n* = 95), prospective-delay (*n* = 115), retrospective-immediate (*n* = 101), and retrospective-delay (*n* = 109). Informed consent was obtained with approval from the Columbia University Institutional Review Board. Participants received a base payment of $12.00 and a bonus payment of up to $15.36, based on their performance on item and source memory tests (96 motivated items * 2 questions (1 item memory question, 1 source memory question) * 8 cents = $15.36).

#### Stimuli.

Participants viewed sequences of grayscale objects (Frank et al., 2020). A total of 288 images were shown to each participant during encoding. Each object image was paired with a colored border (red, purple, blue, or green). Items in each “event” all had the same border color (event structure described below), and the color was chosen pseudorandomly such that consecutive events were assigned different colors.

#### Procedure.

**Figure 1.**
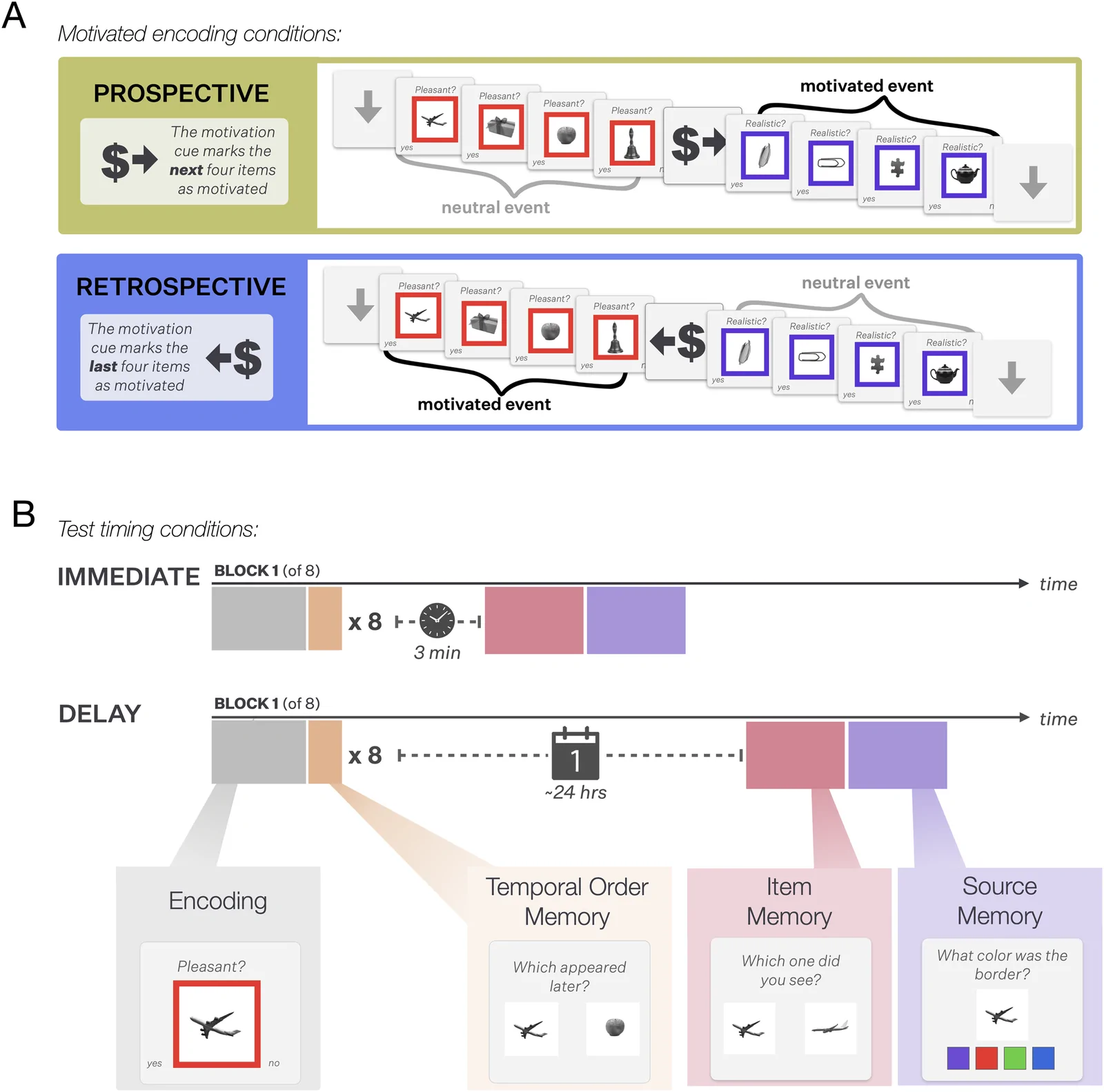
**Experimental paradigm.** A. During each encoding block, 9 four-item “event” sequences of grayscale objects were presented. During each event, items were presented with a colored border and participants were asked to judge whether it would be realistic or pleasant for the object to have this color, with the color and judgment type switching every event. At the boundaries between events, an icon indicated whether the items in an event would yield bonus payments in a later memory test; this information was presented before the motivated event for some participants (prospective condition) and after the motivated events for others (retrospective condition). B. Participants completed 8 encoding blocks during which they viewed sequences of objects pictures. The encoding blocks were interleaved with short temporal order memory test blocks, followed by a delay of either 3 minutes (**immediate condition**) or 24 hours (**delay condition**) before an item memory test and a source memory test.

In this paradigm, participants studied objects and completed three tests, each probing different aspects of episodic memory (see Figure 1). They began by reading task instructions, taking a comprehension quiz to ensure that they understood the instructions, and completing a brief practice round to familiarize themselves with the structure of the task. Next, participants completed 8 blocks of encoding, each followed by a brief temporal order memory test. After the 8 blocks, participants completed the final memory test (see details below).

We used a between-subjects design for our experiment. This design choice was motivated by two considerations. First, given that there are relatively few studies directly comparing prospective and retrospective motivation, we wanted to minimize task complexity for an online sample by ensuring participants only needed to learn one cue-reward contingency. Second, prior work suggests that reward-enhanced memory effects may be attenuated when the proportion of rewarded trials is high (Nagel et al., 2024), so we sought to maintain a relatively low ratio of motivated to neutral trials. A natural follow-up, now that we have established these effects, would be a within-subjects design that will increase this proportion or lengthening the task, to examine the replication of the results we report here. Participants were assigned to one of four conditions (*2 motivation conditions* × *2 test delay conditions*). Participants were assigned to either the **Prospective** motivation condition (in which the motivation cue appears prior to encoding) or the **Retrospective** motivation condition (in which the motivation cue appears *after* encoding). One key difference between the motivation conditions is that participants in the retrospective condition may have experienced greater overall motivation throughout the experiment, since they only learned an event’s reward status after it occurred. However, because motivation effects were analyzed relative to performance on neutral cues, the retrospective results specifically reflect memory enhancement driven by motivational cues rather than a general improvement in memory across the experiment.

Participants were also assigned to one of two test delay conditions. In the *immediate condition*, participants completed item and source memory tests after only three minutes of an even/odd distractor task (see description below). In the *delay condition*, participants completed item and source memory tests approximately 24 hours after encoding (participants completed the encoding phase by midnight EST, and had from 6:30 am to midnight EST the following day to complete the test phase, see Supplementary Figure 1 for a distribution of hours between sessions in the delay condition).

##### Encoding.

For the encoding phase of the task, we adapted a version of the Ezzyat-Dubrow-Davachi paradigm, specifically using a version designed to elicit event boundaries through color and cover task changes (Buonomano et al., 2023; Cowan et al., 2024; Heusser et al., 2018). Participants were shown a series of object images framed by colored borders. Each trial (object and border pair) was shown for 2.5 seconds with a 2-second inter-trial interval. While the object was on screen, participants were instructed to imagine the object on screen in the color of its border, and answer one of two questions: “Is this object-color combination pleasant?” or “Is this object-color combination realistic?.” The task and the border color remained consistent for a sequence of four successive images, constituting an “event.” Colors were pseudo-randomized across events, such that no two consecutive events had the same color. The encoding task (“pleasant?” or “realistic?”) alternated every other event, and the encoding task assigned to the first event was randomly chosen for each participant.

The encoding phase was organized into 8 blocks, and each block contained with 9 events (9 events * 4 items = 36 items per block). The encoding phase included 8 blocks * 36 items per block = 288 objects total. In between each event, participants were shown an arrow cue for 4.5 seconds. Crucially, reward motivation was manipulated within subjects by pseudo-randomly assigning each event to one of two conditions: motivated and neutral. Specifically, in each block, either two, three, or four of nine events were randomly assigned to be “motivated”, ensuring the the number of motivated events was roughly equal across blocks. One third of all events in the encoding phase were assigned to the motivated condition (24 events out of 72). Motivated events are accompanied by a green arrow and dollar sign, and neutral events are accompanied by a black arrow. The timing of the motivation cue depended on whether participants were assigned to the **prospective** or **retrospective** motivation condition. On the subsequent item and source memory tests, participants earned an 8 cent bonus for every correct answer on a trial about a motivated item. Questions about neutral items did not affect participants’ earnings. Participants in the prospective condition were shown the following text during the initial task instructions: “When you see the GREEN CUE it means the NEXT 4 items are REWARDED items. This means that remembering these items when you complete PART 2: memory quiz will earn you bonus money. So pay extra attention to the NEXT 4 items after this cue! For every question you get correct about a REWARDED item, you will get a 8 cent bonus!”. Participants in the retrospective condition received analogous instructions referring to the preceding 4 items. Note that participants did not receive bonus rewards based on their performance on the temporal order memory test.

##### Even/Odd Distractor Task.

After each encoding block, participants completed a brief distractor task (45 seconds long). Participants viewed a stream of numbers and were asked to report if a number was even or odd as quickly as possible. Each number was displayed for 800 ms with a 200 ms inter-trial interval. A three-minute version of this task was also used before the item memory test for the immediate-memory group.

##### Temporal Order Memory Test.

Following the even/odd distractor task, participants were tested on the relative timing of the object stimuli. Participants were shown two objects studied on the preceding block that either belonged to the same event (“within-event”) or two separate events (“across-event”), and were asked to indicate which object appeared later. Each participant completed a total of 32 temporal order memory trials, with an equal split between within-event and across-event trials. Of the 16 within- and 16 across-event trials, half consisted of “lag 2” item pairs, and the other half consisted of “lag 3” item pairs. For “lag 2” and “lag 3” trials, the temporal test items were separated by two or three trials (objects or reward cues) during encoding, respectively. For example, a “lag 3, within-event” trial would include items at positions 1 and 4 within the same event, while a “lag 3, across-event” trial might contain items at position 4 of the first event and position 2 of the next event. During encoding, the duration of the cue trial (the arrow shown between events) was the same as an item trial (the object with a colored border), ensuring that the time interval between item pairs for both within-event and across-event trials was consistent.

##### Item Memory Test.

Participants answered 192 item memory questions in which they had to choose between the item they studied at encoding and a highly similar lure. All motivated items were tested (96 total), and a subset of neutral items were tested (neutral items were chosen such that a roughly equal proportion of neutral items were tested from each block). This test was self-paced, but each trial would end after 15 seconds without a response. After each trial, participants received feedback indicating if their answer was correct, and if they received bonus money (8 cents for every correct answer pertaining to an item in a motivated event). After the item test, participants completed a 192 question source memory test.

##### Source Memory Test.

Participants answered 192 source memory questions, in which they had to select the border color of an item shown at encoding. All motivated items were tested (96 total), and a pseudo-randomly chosen subset of neutral items were tested (same as those used in the item memory test). This test was self-paced, but each trial would end after 15 seconds without a response. After each trial, participants received feedback indicating if their answer was correct, and if they received bonus money (8 cents for every correct answer pertaining to an item in a motivated event).

### Experiment 2

To test the robustness of the results observed in Experiment 1, we conducted a replication experiment (see *attached pre-registration*). The task design was identical to Experiment 1, save three minor changes: 1. The temporal order memory test was modified such that participants were only shown “within” event comparisons, in order to maximize power to detect effects of the motivation manipulation on temporal order memory; 2. We updated the instructions with image diagrams in order to improve clarity and in attempt to decrease attrition due to instruction quiz failure; 3. We added language in the instructions emphasizing that performance on the temporal order test does not affect bonus payment, but reminding participants that below chance performance on any portion of the task would affect payment eligibility overall (specifically the following text was added: “ORDER questions do NOT earn you bonus money, but try your best. Failure to respond or below chance performance on any part of the study affects payment eligibility.”).

#### Participants.

A total of 715 participants were recruited for the Experiment 2 online via the platform Prolific. All individuals included were proficient in English, possessed normal or corrected vision, and normal color vision. Of these participants, 21 did not return for the second part of the study, 39 failed attention checks, 28 missed too many trials (>15% on any given portion of the study), 109 failed one or more comprehension questions, and 35 experienced technical errors. Thus the final sample consisted of 483 participants (246 female; mean age = 25.16 ± 3.4 years, age range = 18–30 years). Participants breakdown by experimental condition was as follows: prospective-immediate (*n* = 130), prospective-delay (*n* = 126), retrospective-immediate (*n* = 113), and retrospective-delay (*n* = 114). Informed consent was obtained with approval from the Columbia University Institutional Review Board. Payment structure was identical to that of Experiment 1.

### Pre-Registered Behavioral Analyses

#### Item Memory.

**Figure 2.**
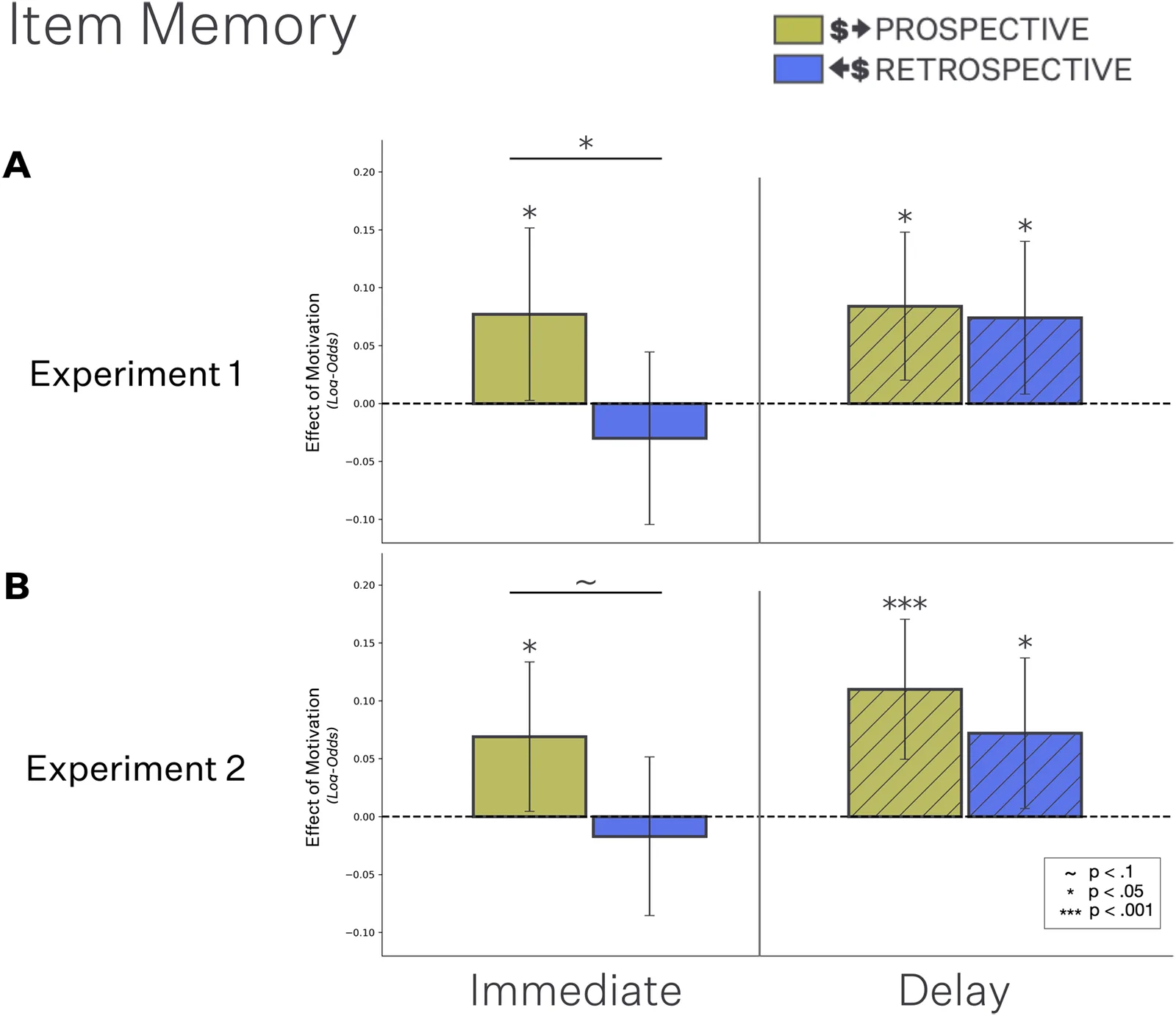
**Effect of motivated encoding condition and test delay on item memory.** Effect of motivation on item memory. Each bar represents the effect size of motivation as determined by a binomial mixed effects model of motivation on memory accuracy. Error bars indicate 95% confidence intervals of the coefficient estimate. While prospective motivation improved memory regardless of consolidation time, retrospective motivation enhanced item memory only after a delay.

To determine the effect of motivation condition on item memory, we conducted a binomial mixed effects model using the package lme4 (Bates et al., 2015) via the Python interface pymer4 (Jolly, 2018). All models were fit at the individual trial level, with accuracy on each trial coded as a binary outcome (correct = 1, incorrect = 0). P-values for fixed effects were derived using Wald z-tests. We performed three versions of this analysis: 1. Separately on each group of subjects (2 motivation conditions × 2 timing conditions). 2. Collapsing across timing conditions, and adding an interaction term for timing. The first method was specified as follows: *correct* ∼ *is_motivated* + (1|*subject*), where *is_motivated* refers to whether an item was in a motivated or neutral event. The second method was specified as follows: *correct* ∼ *is_motivated* * *timing* + (1|*subject*), where *timing* is either immediate or delayed. 3. Collapsing across motivation conditions (prospective and retrospective) to assess the relative effect of the two: *correct* ∼ *motivation_type* * *is_motivated* + (1|*subject*), where *motivation_type* is prospective or retrospective. These analyses were performed two ways: 1. Removing items seen twice due to the temporal order memory task (see Figure 2). 2. Using all items (see Supplementary Figure 2).

#### Source Memory.

To determine the effect of motivation condition on source memory, we conducted a binomial mixed effects model following the same approach described above for item memory. We performed three versions of this analysis: 1. Separately on each group of subjects (2 motivation conditions × 2 timing conditions). 2. Collapsing across timing conditions, and adding an interaction term for timing. The first method was specified as follows: *correct* ∼ *is_motivated* + (1|*subject*), where *is_motivated* refers to whether an item was in a motivated or neutral event. The second method was specified as follows: *correct* ∼ *is_motivated* * *timing* + (1|*subject*), where *timing* is either immediate or delayed. 3. Collapsing across motivation conditions (prospective and retrospective) to assess the relative effect of the two: *correct* ∼ *motivation_type* * *is_motivated* + (1|*subject*), where *motivation_type* is prospective or retrospective. These analyses were performed two ways: 1. Removing items seen twice due to the temporal order memory task (see Figure 4). 2. (see Supplementary Figure 3).

#### Temporal Order Memory.

To ensure that the paradigm successfully induced event boundaries, we sought to replicate the finding that accuracy for temporal order memory is higher within events. To test this, we conducted a linear mixed effects model assessing the effect of comparison type (within-event vs. across-event) on temporal memory accuracy for Experiment 1. The model was specified as follows: *correct* ∼ *comparison_type* + (1|*subject*). As with the item and source memory analyses, models were fit at the individual trial level with binary accuracy as the outcome, and *p*-values were derived using Wald *z*-tests.

Next, we wanted to investigate if the motivated encoding manipulation impacted temporal order memory. Due to a limited number of temporal order trials, and the subsequent removal of “across-event” temporal order trials in Experiment 2 (to maximize power), we subset the data to “within-event” trials. We then assessed if temporal memory was improved for motivated events compared to neutral events. The model was specified as follows: *correct* ∼ *is_motivated* * *motivation_condition* + (1|*subject*), where *motivation_condition* refers to whether the participant was assigned to the prospective or retrospective condition.

### Exploratory Behavioral Analyses

#### Carry-Over Effects of Motivation.

To explore the specificity of motivation’s benefit, we examined if items proximal to a motivational cue are better remembered, even when they are not pertinent to the monetary bonus. To test this, we conducted a model of memory accuracy specified as follows: *accuracy* ∼ *reward_cue_before* * *reward_cue_after*, where *reward_cue_before* refers to whether or not the items were preceded by a reward cue, and *reward_cue_after* refers to whether items were followed by a reward cue. The interaction revealed the effect of having a reward cue preceding and following a given event. The reference level consisted of events that were preceded and followed by neutral cues.

#### Event Position and Memory.

We tested if item memory varied as a function of which part of an event an item came from. For instance, do we observe a boost in memory just prior to event boundaries? To investigate this, we ran the following model for both item and source memory: *correct* ∼ *event_index* + (1|*subject*).

**Figure 3.**
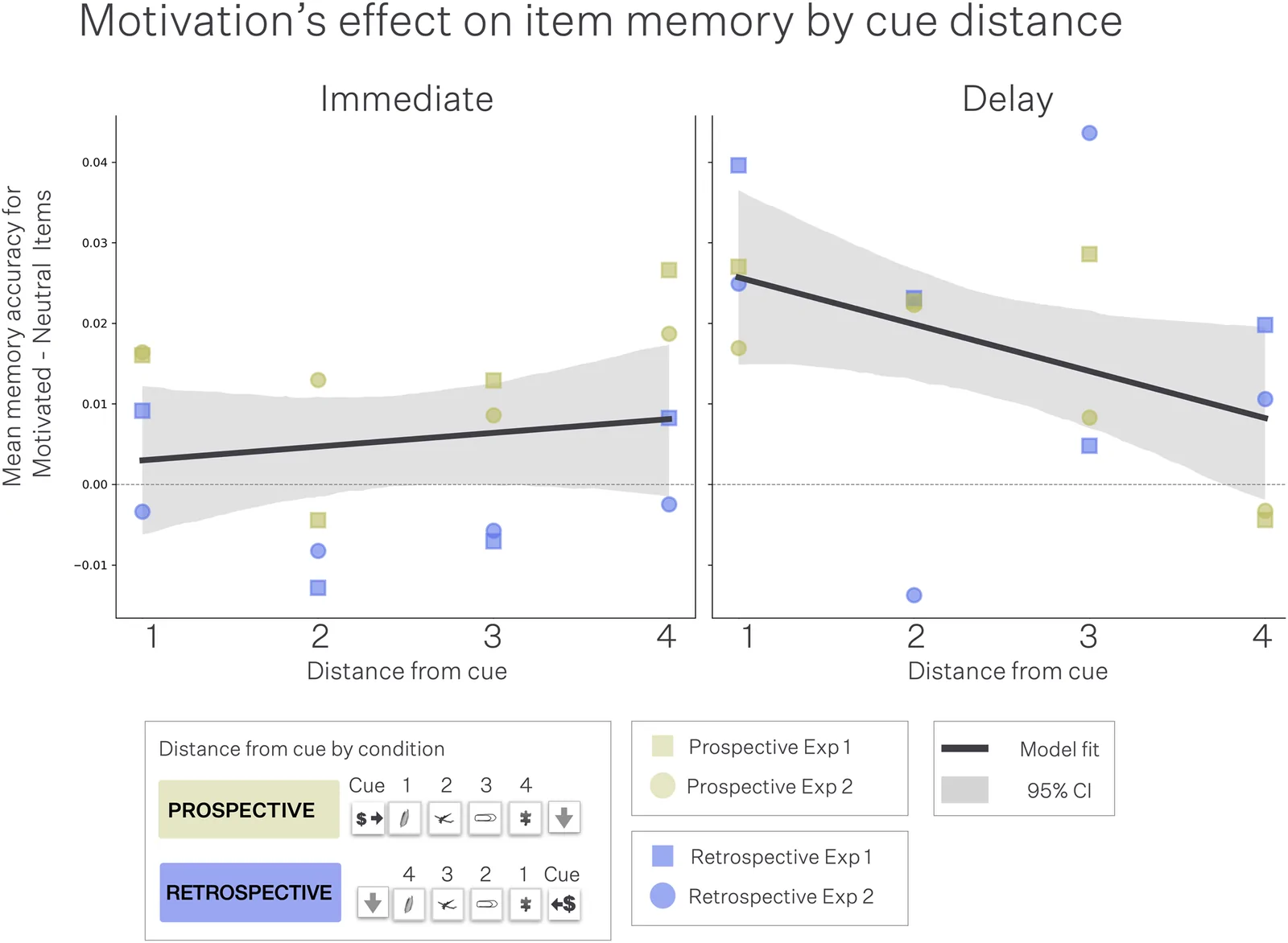
**Effect of motivation on item memory by distance from motivational cue.** Colored markers show the average difference in memory accuracy for motivated vs. neutral items, according to the item’s distance from the cue (a distance of 1 is the item adjacent to the cue, a distance of 4 is the item farthest from the cue). Black lines show the model fit (‘accuracy_difference ∼ distance_from_cue’). Green and blue markers represent values for prospective and retrospective conditions, respectively; squares denote values for Experiment 1 while circles denote values for Experiment 2. We see no significant effect of distance in item memory measured immediately, but item memory measured after a delay shows a negative effect, such that the influence of motivation decreases with distance from the cue.

Next we asked if there was an interaction between motivation’s modulation of memory and an item’s position in an event. To test if motivational enhancement scales linearly with distance from the motivational cue, we ran a separate model, including cue_distance as a continuous variable, repeated for both item and source memory: *correct* ∼ *cue_distance* * *is_motivated* + (1|*subject*). To better visualize the effect of *cue_distance*, we ran this model at the subject level: *motivation_effect* ∼ *cue_distance*, where *motivation_effect* is the average difference in accuracy for motivated vs. neutral items for each subject (see Figure 3).

### Transparency and Openness

Experiment 2 task, primary analyses, and exclusion criteria were pre-registered at https://aspredicted.org/vnys-yxm7.pdf. In our pre-registration, we wrote that we aimed to collect 150 participants per condition. Due to a bug in the code used to monitor data collection, we collected a greater number of participants than intended, yielding a final sample size of 715 (i.e., approximately 178 participants per condition prior to exclusion). Additionally, in our preregistered analyses, we specified that the analyses of motivation would be conducted with mixed effects models that included the following random effects: (1 + is_motivated|subject) + (1|item). However, due to convergence errors that indicated our model was over-specified, we simplified the random effects, instead choosing to include only a random intercepts for subject: (1|subject). Finally, in the pre-registration we did not specify if the models would be run on the data from Experiments 1 and 2 separately or combined. Here we chose to report all three versions (Experiments 1 and 2 separately, as well as across the full sample) to show which effects replicate across experiments as well as summarize the effect across both studies. Code and data will be made available upon publication of the manuscript at https://github.com/dpmlab/motivated_memory.

## RESULTS

### For Stimuli Earlier in an Event, Item Memory Is Enhanced, While Source Memory Is Impaired

Before assessing how motivation modulated item, source, and temporal order memory, we first examined memory performance generally. Performance on the item memory test was largely above chance with 99.77% of participants performing above 50% in the immediate condition, and 97.84% in the delay condition. Performance did not vary significantly across Experiments 1 and 2 for the immediate condition (Experiment 1: *M* = 0.75, *SD* = 0.07, *N* = 196; Experiment 2: *M* = 0.74, *SD* = 0.08, *N* = 243; *t*(437) = 1.4, *p* = 0.16) or the delay condition (Experiment 1: *M* = 0.68, *SD* = 0.08, *N* = 224; Experiment 2: *M* = 0.68, *SD* = 0.08, *N* = 240; *t*(462) = 0.25, *p* = 0.8). Performance on the source memory test was also largely above chance (25%), with 1.82% falling below chance in the immediate condition, and 6.47% falling below chance in the delay condition. Accuracy did not vary significantly across Experiments 1 and 2 for either the immediate condition (Experiment 1: *M* = 0.46, *SD* = 0.12, *N* = 196; Experiment 2: *M* = 0.45, *SD* = 0.12, *N* = 243; *t*(437) = 1.3, *p* = 0.19) or the delay condition (Experiment 1: *M* = 0.35, *SD* = 0.08, *N* = 224; Experiment 2: *M* = 0.36, *SD* = 0.09, *N* = 240; *t*(462) = −1.25, *p* = 0.21).

The temporal order memory test was the most challenging for participants, but 88.04% of participants performed above 50% (chance level performance for a two-alternative forced choice test) and overall performance was similar to previous studies using a highly similar task (Cowan et al., 2024). Performance was slightly better on this task in Experiment 2 compared to Experiment 1(Experiment 1: *M* = 0.63, *SD* = 0.14, *N* = 196; Experiment 2: *M* = 0.65, *SD* = 0.13, *N* = 243; *t*(901) = −2.64, *p* = 0.008). To confirm that the paradigm produces event boundaries as intended, we replicated past work showing greater temporal order memory within vs. across events (see Supplementary Figure 4). We note that our paradigm may produce stronger event boundary effects than typical Ezzyat–DuBrow–Davachi designs, given that the inter-event interval in our task is longer and involves an additional button press, both of which could reinforce the formation of discrete events. See supplement for visualization of overall memory accuracy hit rate (Supplementary Figure 5) and for item and source hit-rate grouped by condition (item: Supplementary Figure 6, source: Supplementary Figure 7).

As an exploratory analysis, we investigated the impact of event position on memory, independent of motivation, to determine if memory is better for information that occurred at the start or the end of an episodic event. Prior research has reported mixed results regarding the impact of event boundaries on item memory. Some studies show enhanced recognition memory for items encoded concurrent with a boundary (McClay et al., 2023; Swallow et al., 2009). In contrast, other work reports no difference in recognition memory for items appearing before versus after a boundary (DuBrow & Davachi, 2013), while still other studies find improved item memory (better discrimination from similar lures) for items presented immediately *before* a boundary (Morse et al., 2023). Looking first at item memory, we observe a significant effect of position, such that item memory is improved for items occurring later in an event (Experiment 1, immediate: *β* = 0.04, 95% CI [0.02, 0.06], *p* < .001; Experiment 1, delay *β* = 0.02, 95% CI [0.0, 0.04], *p* = 0.027; Experiment 2, immediate: *β* = 0.03, 95% CI [0.01, 0.05], *p* = 0.004; Experiment 2, delay *β* = 0.04, 95% CI [0.02, 0.05], *p* < .001; Combined sample, immediate: *β* = 0.03, 95% CI [0.02, 0.05], *p* < .001; Combined sample, delay: *β* = 0.03, 95% CI [0.02, 0.04], *p* < .001; see Supplementary Figure 8, for results split by motivated vs. neutral events see Supplementary Figure 9).

Interestingly, source memory shows the opposite pattern, such that accuracy is greater for items occurring earlier in the event. We observe a negative relationship between event index and source memory across experiments and delay conditions, and the effect is significant in all but the Experiment 1 delay condition (Experiment 1, immediate: *β* = −0.03, 95% CI [−0.05, −0.01], *p* = 0.001; Experiment 1, delay *β* = −0.01, 95% CI [−0.03, 0.01], *p* = 0.18; Experiment 2, immediate: *β* = −0.04, 95% CI [−0.06, −0.02], *p* < .001; Experiment 2, delay *β* = −0.04, 95% CI [−0.05, −0.02], *p* < .001; Combined sample, immediate: *β* = −0.04, 95% CI [−0.05, −0.02], *p* < .001; Combined sample, delay: *β* = −0.02, 95% CI [−0.04, −0.01], *p* < .001; see Supplementary Figure 8, for results split by motivated vs. neutral events see Supplementary Figure 9). This finding replicates prior work showing improved source memory for information encountered early in an event, after an event boundary (Clewett et al., 2020; Cowan et al., 2024; Heusser et al., 2018). Taken together, these results reveal a double dissociation: item memory benefits from later event positions, whereas source memory benefits from earlier ones.

### Episodic Memory Is Consistently Enhanced by Prospective Motivation, While Retrospective Motivation Effects May Require Consolidation

#### Item Memory.

In Experiment 1, item memory was significantly enhanced by prospective motivation for participants in the immediate condition (*β* = 0.08, 95% CI [0.0, 0.15], *p* = 0.043; see Figure 2A). This effect replicated in Experiment 2 (*β* = 0.07, 95% CI [0.0, 0.13], *p* = 0.036; see Figure 2B), and was significant in the combined sample (*β* = 0.07, 95% CI [0.02, 0.12], *p* = 0.004). For participants in the delay condition, memory for motivated items was significantly better than for neutral items in Experiment 1 (*β* = 0.08, 95% CI [0.02, 0.15], *p* = 0.011; see Figure 2A) and Experiment 2 (*β* = 0.11, 95% CI [0.05, 0.17], *p* < .001; see Figure 2B), as well as in the combined sample (*β* = 0.1, 95% CI [0.05, 0.14], *p* < .001). Altogether, pre-encoding motivation reliably enhanced item memory, regardless of the time participants have for memory consolidation.

In contrast to prospective motivation, the efficacy of retrospective motivation on item memory was contingent on consolidation time. Across both experiments, there was no effect of retrospective motivation memory in the immediate condition (Experiment 1: *β* = −0.03, 95% CI [−0.1, 0.04], *p* = 0.42; Experiment 2: *β* = −0.02, 95% CI [−0.09, 0.05], *p* = 0.62; see Figure 2). Additionally, in both experiments, the immediate effect of retrospective motivation was weaker than that of prospective motivation, and this difference was significant in the first experiment (Experiment 1: *β* = −0.11, 95% CI [−0.21, −0.0], *p* = 0.046; Experiment 2 (*β* = −0.09, 95% CI [−0.18, 0.01], *p* = 0.07; see Figure 2). On the other hand, in the delay condition of Experiment 1, we observed a significant effect of retrospective motivation (*β* = 0.07, 95% CI [0.01, 0.14], *p* = 0.028; see Figure 2A), and this dissociation between immediate and delayed test replicated in Experiment 2 (immediate condition: *β* = −0.02, 95% CI [−0.09, 0.05], *p* = 0.62; delay condition: *β* = 0.07, 95% CI [0.01, 0.14], *p* = 0.03; see Figure 2B), as well as in the combined sample (immediate: *β* = −0.02, 95% CI [−0.07, 0.03], *p* = 0.36; delay: *β* = 0.07, 95% CI [0.03, 0.12], *p* = 0.002). Using a model on data from both delay conditions, we observed a significant interaction of motivation type and delay condition in Experiment 1 (*β* = −0.11, 95% CI [−0.2, −0.02], *p* = 0.03), although this effect was only marginally significant in Experiment 2 (*β* = −0.09, 95% CI [−0.16, 0.01], *p* = 0.064). This effect was significant when combining samples from Experiments 1 and 2 (*β* = −0.096, 95% CI [−0.15, −0.03], *p* = 0.006). These results of the retrospective condition data suggest items can be successfully prioritized in memory after encoding, but that effect depends on consolidation.

While the prior results speak to the impact of the cue on the items designated as “motivated”, we also wanted to examine the specificity of the motivational cue. For instance, do the events preceding a prospective cue or the events following a retrospective cue also receive a memory boost due to their proximity to the reward cue, despite the fact that memory for these events do not affect bonus payment? To test this hypothesis, we ran a model assessing the impact of event status on memory, comparing accuracy for stimuli in events falling after a reward cue, before a reward cue, as well as the interaction of these factors (i.e. events flanked by reward cues). However, mere proximity to the reward cue did not significantly enhance memory across in any of the four conditions tested (prospective immediate, prospective delayed, retrospective immediate, retrospective delayed: all *p*’s > .05). Consequently, the benefit of the motivational cue on item memory does not appear to extend beyond the actual stimuli cued as rewarded (see Supplementary Figure 10).

In addition to exploring the impact of motivation on item memory overall, we were also curious about potential interactions between event index and the effect of motivated encoding on memory. One possibility is that motivation enhances memory in a graded fashion, with items closer to the motivation cue demonstrating the largest benefit, with a decreasing effect the farther the item is from the cue. Alternatively, one might predict that items are bound strongly into a singular event unit, either due to implicit processing or conscious strategies employed during encoding. If motivation operates on the event as a unit, one might anticipate uniform enhancement of items at all positions.

To test the hypothesis that motivation enhances memory in a graded fashion, we added an interaction term for cue distance into our model. We see no evidence of an interaction between motivation and cue distance in the intermediate condition. However in the delay condition, across both prospective and retrospective motivation conditions, the interaction between distance from cue and motivation is negative (prospective: *β* = −0.04, 95% CI [−0.07, −0.0], *p* = 0.034; retrospective: *β* = −0.02, 95% CI [−0.05, 0.02], *p* = 0.38; combined *β* = −0.03, 95% CI [−0.05, −0.0], *p* = 0.033). Stated another way, the impact of motivation is larger for items closer in time to the moment of the motivation cue (items at the beginning of prospectively motivated events and at the end of retrospectively motivated events, see Figure 3).

#### Source Memory.

**Figure 4.**
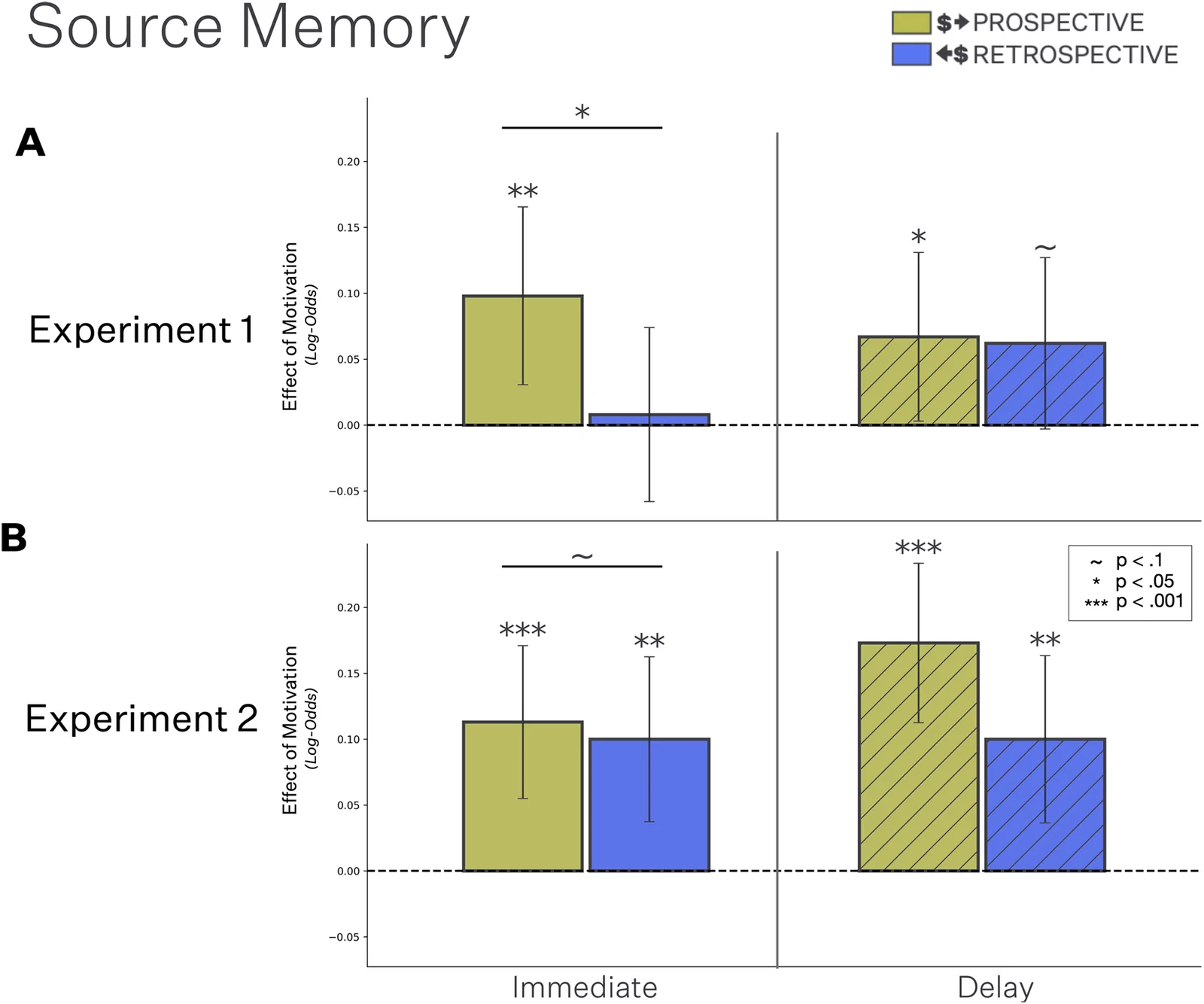
**Effect of motivated encoding condition and test delay on source memory.** Effect of motivation on source memory. Each bar represents the effect size of motivation as determined by a binomial mixed effects model of motivation on memory accuracy. Error bars indicate 95% confidence intervals of the coefficient estimate.

Prospective motivation significantly enhanced source memory in both the immediate and delay condition in Experiment 1 (**immediate**: *β* = 0.1, 95% CI [0.03, 0.17], *p* = .004; **delay**: *β* = 0.07, 95% CI [0.01, 0.13], *p* = 0.39; see Figure 4A), and both of these effects replicated in Experiment 2 (**immediate**: *β* = 0.11, 95% CI [0.06, 0.17], *p* < .001; **delay**: *β* = 0.17, 95% CI [0.11, 0.23], *p* < .001; see Figure 4B), as well as in the combined sample (**immediate**: *β* = 0.11, 95% CI [0.06, 0.15], *p* < .001; **delay**: *β* = 0.12, 95% CI [0.08, 0.17], *p* < .001).

Similar to the pattern observed in item memory, in Experiment 1 retrospective motivation had no effect on source memory in the immediate condition, but marginally benefited memory in the delay condition (**immediate**: *β* = .008, 95% CI [−0.06, 0.07], *p* = 0.82; **delay**: *β* = 0.06, 95% CI [0.0, 0.13], *p* = 0.06; see Figure 4A). However, contrary to our pre-registered prediction, this pattern did not replicate in Experiment 2. Rather, source memory was better for motivated items compared to neutral items for participants in both delay conditions (**immediate**: *β* = 0.1, 95% CI [0.038, 0.163], *p* = .002; **delay**: *β* = 0.1, 95% CI [0.037, 0.164], *p* = .002; see Figure 4B). Combining samples from Experiments 1 and 2, the effect of retrospective motivation on source memory is significant in both delay conditions (**immediate**: *β* = 0.08, 95% CI [0.04, 0.13], *p* = .015; **delay**: *β* = 0.08, 95% CI [0.04, 0.13], *p* < .001).

To understand the specificity of the motivation’s benefit on source memory, we examined if items proximal to a motivational cue are better remembered, even when they are not pertinent to the monetary bonus. To assess this, we compared source memory accuracy for events that fell before a reward cue, after the reward cue, as well as the interaction of reward-before and reward-after conditions, relative to neutral-flanked stimuli. For the immediate condition, mere proximity to a reward cue did not significantly enhance memory (all *p*s > .05), or for the prospective delayed condition (Experiment 1: *β* = 0.016, 95% CI [−0.016, 0.047], *p* = 0.323; Experiment 2: *β* = −0.002, 95% CI [−0.036, 0.031], *p* = 0.888; Combined: *β* = 0.006, 95% CI [−0.016, 0.029], *p* = 0.583). However after a delay, there was a significant effect of proximity to reward in the retrospective condition combined sample such that events falling *after* the motivational cue were better remembered, relative to neutral-flanked events (Experiment 1: *β* = 0.025, 95% CI [−0.007, 0.056], *p* = 0.129; Experiment 2: *β* = 0.023, 95% CI [−0.009, 0.055], *p* = 0.153; Combined: *β* = 0.024, 95% CI [0.001, 0.047], *p* = 0.037). Consequently, after a 24-hour delay, the benefit of retrospective motivation may extend forward to reward-irrelevant items (see Supplementary Figure 11).

Finally, we assessed if motivation’s impact on source memory depends on distance from the motivational cue. To determine if motivation enhances source memory in a graded fashion, we added an interaction term for cue distance into our model of memory. Source memory demonstrates a qualitatively similar pattern to item memory (wherein, after a delay, distance from the cue is negatively correlated with motivation’s effect), but these results are not significant (all *p*s > .05; see Supplementary Figure 12).

### Prospective and Retrospective Motivated Encoding Have Opposing Effects on Temporal Order Memory

To evaluate the impact of our motivated encoding manipulation on temporal order memory, we analyzed within-event trials, testing whether participants were more accurate at judging the relative order of two items from the same event when that event was motivated versus neutral (see Methods for details). While we hypothesized an effect of motivation on item memory prior to running Experiment 1 and 2, for temporal order memory, the expected effect of motivation seemed less clear. On the one hand, participants were not rewarded for their performance on this task and therefore might be expected to show no improvement for motivated events. Alternatively, temporal order memory is thought to reflect the extent to which discrete stimuli are linked into a single representation (Clewett & Davachi, 2017; Clewett et al., 2019; DuBrow & Davachi, 2013, 2016; Ezzyat & Davachi, 2011), and could be expected to be larger for events in which participants are motivated to remember the shared context for all items.

**Figure 5.**
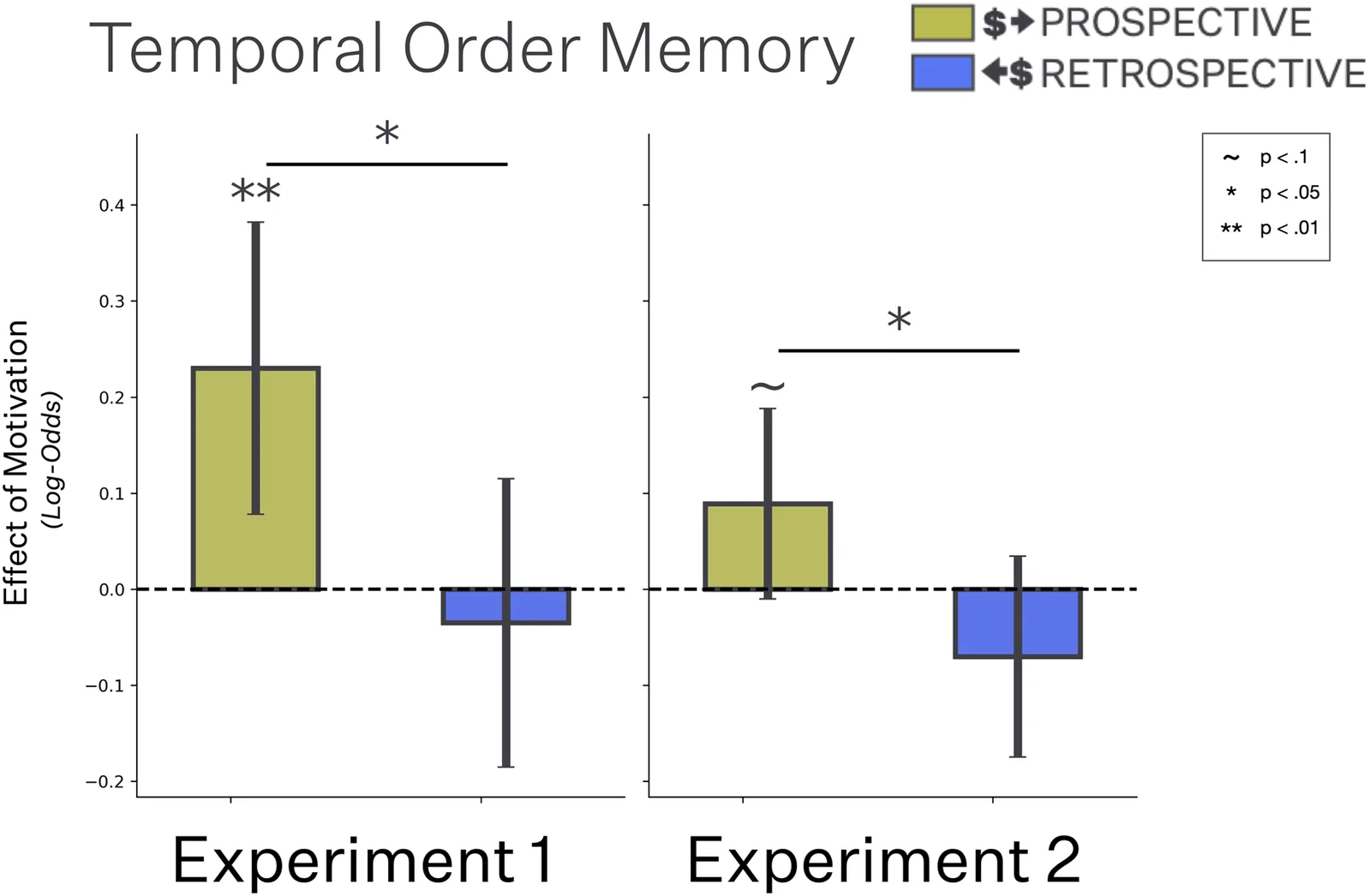
**Effect of motivated encoding condition on temporal order memory.** Effect of motivated encoding condition on temporal order memory for within-event trials. Bars represent the effect size (log-odds coefficient) of motivation on temporal order accuracy, as determined by a binomial mixed effects model predicting whether participants correctly identified which of two items from the same event appeared later. Error bars indicate 95% confidence intervals of the coefficient estimate. Comparisons between bars test the significance of the interaction between motivation status (motivated vs. neutral) and motivation condition (prospective vs. retrospective). *Note*: this analysis collapses across delay conditions, as temporal order memory always occurs during encoding.

In both experiments, we found that prospective and retrospective conditions had significantly different relationships between event motivation and temporal order memory (Experiment 1: *β* = −0.26, 95% CI [−0.48, −0.05], *p* = 0.015; Experiment 2: *β* = −0.16, 95% CI [−0.3, −0.02], *p* = 0.031; see Figure 5), and this effect was also observed when combining experiments (*β* = −0.19, 95% CI [−0.31, −0.07], *p* = 0.002). Prospective motivation improved temporal order memory, and this effect was significant in Experiment 1, marginal in Experiment 2, and significant when combining experiments (Experiment 1: *β* = 0.23, 95% CI [0.08, 0.38], *p* = 0.003; Experiment 2: *β* = 0.09, 95% CI [−0.01, 0.19], *p* = 0.08; Combined: *β* = 0.13, 95% CI [0.05, 0.21], *p* = 0.002). Retrospective motivation numerically *decreased* temporal order accuracy in both experiments, though this effect was not significant (Experiment 1: *β* = −0.04, 95% CI [−0.18, 0.12], *p* = 0.64; Experiment 2: *β* = −0.07, 95% CI [−0.18, 0.03], *p* = 0.19; Combined: *β* = −0.06, 95% CI [−0.14, 0.03], *p* = 0.17). Note that in Experiment 2, the instructions to participants emphasized that bonus payments would be unaffected by the temporal order memory test; this may have led to a less robust effect of prospective motivation on accuracy (compared to Experiment 1), but this change did not eliminate the effect of motivation condition type.

## DISCUSSION

The information we encode can differ both in terms of its future value, and when we learn of its future value. There may be cues available at the start of an event that alert us to its significance, but we often don’t learn of an event’s importance until after it ends and we observe an outcome or reward. Here we investigated how motivation timing and consolidation interact to determine what survives in memory. In two experiments, we found evidence that both pre- and post-encoding motivation can enhance later memory. Critically, however, the role of consolidation differed for motivational cues presented before versus after event encoding. Pre-encoding motivation led to an immediate enhancement of item memory, while the effect of post-encoding only emerged after a 24-hour delay. Additionally, we observed that, following a 24-hour delay, the motivational effect was stronger for items closer to the cue. Lastly, the results of a temporal order task revealed that only pre-encoding motivation improved memory for how items arranged within events. Together, our findings suggest that the timing of motivation relative to an event may determine the mechanism by which it affects memory.

In investigating the mechanisms by which motivation shapes memory formation, we sought to adjudicate between two key hypotheses. The first posits that post-encoding motivation, much like receipt of reward at the end of an episode, relies on an extended consolidation period to influence memory. The second hypothesis is that both pre- and post-encoding motivation can affect memory immediately and via similar mechanisms. Our item memory results support the former account, indicating that retroactive motivation requires a consolidation-based mechanism to modulate memory. The source memory results are also largely inline with this interpretation: the benefit of retroactive motivation was more consistent across experiments in the delay condition than in the immediate condition. Finally, results from the temporal memory task provide converging evidence that pre- and post-encoding motivation modulate memory through distinct processes. Prior work has shown that temporal order memory relies on the strength of associative links between items, and that disrupting associative encoding—such as task switches (Wang & Egner, 2022), interleaved distractor tasks (DuBrow & Davachi, 2013), and context changes in color or judgment type that induce event boundaries (Cowan et al., 2024; Heusser et al., 2018)—impairs temporal memory. These findings align with the interpretation that pre-encoding motivation enhances within-event binding, thereby strengthening associative memory. However, as our current design assesses memory only on the day of encoding, further research is required to determine whether post-encoding motivation fails to enhance memory due to the necessity of anticipatory processes during encoding, or whether retroactive effects might emerge given sufficient consolidation time. It should be noted that performance on the temporal memory test is quite low, both in the present study and in past research using a similarly structured task (Cowan et al., 2024). As a result, temporal order memory may need to be operationalized differently in tasks assessed after a 24-hour delay to prevent floor effects.

Past research has investigated the cognitive mechanisms underlying motivated memory by contrasting behavior and brain activity for neutral and motivated stimuli (Adcock et al., 2006; Cohen et al., 2014; Elliott & Brewer, 2019; Poh et al., 2022; Wolosin et al., 2012). Expanding on this work, the results of two complementary measures of memory studied here highlight that the behavioral effects of motivation are also critically dependent on its timing relative to encoding. More broadly, our findings argue that distinctions in memory processes should not be drawn solely based on motivational relevance (i.e., motivated vs. neutral), but must also consider the temporal dynamics of motivation signals (i.e., pre- vs. post-encoding). In this sense, identifying when motivation exerts its influence is essential for carving memory processes at their natural joints, helping to delineate underlying mechanisms that might otherwise be conflated.

Our findings suggest that post-encoding motivation may operate similarly to other types of salience during encoding (e.g., emotional arousal, incidental reward) in that it requires consolidation to improve memory for preceding stimuli (Braun et al., 2018; Dunsmoor et al., 2015, 2022; Patil et al., 2016). A key question that remains, however, is the nature of the consolidation-dependent process driving these effects. One potential mechanism is hippocampal replay, which occurs during sleep and periods of rest and is thought to strengthen the memory traces it reactivates (Atherton et al., 2015; Foster & Wilson, 2006; Lee & Wilson, 2002). Furthermore, reward drives higher rates of reverse replay (Ambrose et al., 2016; Gomperts et al., 2015; Singer & Frank, 2009), offering a route by which neutral memories leading up to a rewarding event might be strengthened over the course of consolidation. Finally, consistent with the graded effect of motivation observed here, rodent research demonstrates that reward-sensitive ventral tegmental area (VTA) neurons coordinate replay, resulting in increased rates of hippocampal replay for locations proximal to reward (Gomperts et al., 2015). Although we test the effect of motivation, rather than direct receipt of reward, prior fMRI research in humans demonstrates that motivational cues also modulate VTA activity (Adcock et al., 2006; Wolosin et al., 2012). Taken together, these studies point to VTA-mediated replay as a plausible mechanistic account of our findings. An important limitation of our design, however, is that our 24-hour delay condition cannot isolate the specific contribution of sleep, as it necessarily includes both sleep and waking periods. A sleep/wake design (e.g., where the delay duration is held constant but only one group sleeps during the delay) could help disentangle this could clarify whether sleep plays a privileged role in consolidating retrospectively motivated memories, or whether the benefit accrues more gradually over time.

While our results align with previous research showing consolidation-dependent retroactive effects, they also raise the question: is consolidation always required for post-encoding motivation to influence memory? Alternatively, the impact of post-encoding cues might depend on their exact timing. The event segmentation literature demonstrates a sharp increase in hippocampal activity at boundaries that scales with event salience and predicts subsequent recall (Baldassano et al., 2017; Barnett et al., 2022; Ben-Yakov & Dudai, 2011; Ben-Yakov & Henson, 2018). Boundary activity has been shown to quickly reactivate representations of the prior event (Silva et al., 2019, 2023; Sols et al., 2017), potentially analogous to replay observed during brief periods of rest during exploration in rodents and nonhuman primates (Carr et al., 2011; Leonard et al., 2015). As a result, it is possible that in our task the post-encoding cue arrived too late (i.e. after the hippocampal boundary activity), whereas a cue presented slightly earlier might yield immediate memory improvements, as seen in the pre-encoding motivation condition.

Aside from the precise timing of the retroactive motivational cue, the content of a memory might also impact whether consolidation is necessary for retroactive effects. Previous research has shown same-day effects of post-encoding value cues on memory for words and sentences (Chung & Federmeier, 2023; Hayes et al., 2013; Villaseñor et al., 2021). One potential explanation for the discrepancy between these studies and our own is stimulus format, and particularly whether that format facilitates rehearsal. Word stimuli may be easier to rehearse than the images in our task, allowing for an immediate retroactive boost in memory that would otherwise require consolidation time.

Rehearsal might also explain why the impact of retrospective motivation was apparent immediately in Experiment 2 for source memory, but was never observed in the immediate condition for item memory. In the item memory task, participants needed to remember the fine-grained details of an item to correctly distinguish it from the lure. Consequently, it might be difficult or impossible to improve one’s accuracy on the item memory test through explicit rehearsal. The source test, on the other hand, required participants only to recall a color and item combination, which might be more conducive to explicit rehearsal. Indeed, some participants reported using such strategies in the experiment feedback form (“repeating in my head what the object was and the color (ex: blue sharpie)”). If rehearsal results in an immediate effect of retrospective motivation, and participants differ in the extent to which they rehearsed, this could explain why we observed inconsistent effects of motivation on source memory across experiments. Future research is needed to clarify the role of rehearsal and determine the contexts in which consolidation is necessary for retroactive memory enhancement.

The type of boundary may also play a role in determining the effect of retrospective motivation. In our paradigm, boundaries were defined by both color and task switches, which prior work suggests elicit robust event boundary effects (Cowan et al., 2024; Heusser et al., 2018). It remains possible that the retrospective cue’s effect depends in part on boundary-driven reactivation of the preceding event, and might differ as a function of how boundaries are defined (i.e., whether boundaries arise from a longer inter-trial interval alone, a task switch, a change in stimulus features, or some combination of these). A design that parametrically varies boundary type or strength while holding cue timing constant could help clarify this relationship.

The role of strategic attention in the prospective condition merits further investigation, considering the potential increased attention that the reward cues may elicit. In the current between-subjects design, participants in the prospective condition can anticipate which events are motivated, potentially allowing them to strategically allocate attention toward motivated events. This is a meaningful feature of prospective motivation as it operates in the real world: we often adjust our encoding effort when we know something is important in advance. However one drawback of this choice is the inability to distinguish between a purely motivational enhancement of memory versus an impairment in memory for neutral items (compared to performance if no cues were provided). A within-subjects design in which participants experience both prospective and retrospective cues would make the upcoming cue type unpredictable, removing the strategic option to pay less attention to neutral events. Likewise, replacing the current reward/no-reward contrast with high and low reward cues could provide incentive to attend across both conditions. Whether the prospective advantage persists under these conditions remains an open question.

In sum, our results reveal dissociable effects of pre- and post-encoding motivation on memory. These findings highlight the critical role of the timing of motivation and provide evidence that multiple mechanisms contribute to value-based memory prioritization.

## AUTHOR CONTRIBUTIONS

T.A.C.: Conceptualization; Data curation; Formal analysis; Investigation; Methodology; Software; Visualization; Writing – original draft. D.S.: Conceptualization; Funding acquisition; Methodology; Project administration; Resources; Supervision; Writing – review & editing. C.B.: Conceptualization; Methodology; Project administration; Resources; Supervision; Writing – review & editing.

## DATA AVAILABILITY STATEMENT

Code and data will be made available upon publication of the manuscript at https://github.com/dpmlab/motivated_memory.

## Supplementary Material



## References

[bib1] Adcock, R. A., Thangavel, A., Whitfield-Gabrieli, S., Knutson, B., & Gabrieli, J. D. E. (2006). Reward-motivated learning: Mesolimbic activation precedes memory formation. Neuron, 50(3), 507–517. 10.1016/j.neuron.2006.03.036, 16675403

[bib2] Ambrose, R. E., Pfeiffer, B. E., & Foster, D. J. (2016). Reverse replay of hippocampal place cells is uniquely modulated by changing reward. Neuron, 91(5), 1124–1136. 10.1016/j.neuron.2016.07.047, 27568518 PMC6013068

[bib3] Atherton, L. A., Dupret, D., & Mellor, J. R. (2015). Memory trace replay: The shaping of memory consolidation by neuromodulation. Trends in Neurosciences, 38(9), 560–570. 10.1016/j.tins.2015.07.004, 26275935 PMC4712256

[bib4] Baldassano, C., Chen, J., Zadbood, A., Pillow, J. W., Hasson, U., & Norman, K. A. (2017). Discovering event structure in continuous narrative perception and memory. Neuron, 95(3), 709–721. 10.1016/j.neuron.2017.06.041, 28772125 PMC5558154

[bib5] Barnett, A. J., Nguyen, M., Spargo, J., Yadav, R., Cohn-Sheehy, B. I., & Ranganath, C. (2022). Dynamic hippocampal-cortical interactions during event boundaries support retention of complex narrative events. bioRxiv. 10.1101/2022.10.23.513391 37944517

[bib6] Bates, D., Mächler, M., Bolker, B. M., & Walker, S. C. (2015). Fitting linear mixed-effects models using lme4. Journal of Statistical Software, 67(1), 1–48. 10.18637/jss.v067.i01

[bib7] Ben-Yakov, A., & Dudai, Y. (2011). Constructing realistic engrams: Poststimulus activity of hippocampus and dorsal striatum predicts subsequent episodic memory. Journal of Neuroscience, 31(24), 9032–9042. 10.1523/JNEUROSCI.0702-11.2011, 21677186 PMC6622928

[bib8] Ben-Yakov, A., & Henson, R. N. (2018). The hippocampal film editor: Sensitivity and specificity to event boundaries in continuous experience. Journal of Neuroscience, 38(47), 10057–10068. 10.1523/JNEUROSCI.0524-18.2018, 30301758 PMC6246887

[bib9] Braun, E. K., Wimmer, G. E., & Shohamy, D. (2018). Retroactive and graded prioritization of memory by reward. Nature Communications, 9(1), 4886. 10.1038/s41467-018-07280-0, 30459310 PMC6244210

[bib10] Buonomano, D. V., Buzsáki, G., Davachi, L., & Nobre, A. C. (2023). Time for memories. Journal of Neuroscience, 43(45), 7565–7574. 10.1523/JNEUROSCI.1430-23.2023, 37940593 PMC10634580

[bib11] Carr, M. F., Jadhav, S. P., & Frank, L. M. (2011). Hippocampal replay in the awake state: A potential substrate for memory consolidation and retrieval. Nature Neuroscience, 14(2), 147–153. 10.1038/nn.2732, 21270783 PMC3215304

[bib12] Chung, Y. M. W., & Federmeier, K. D. (2023). Read carefully, because this is important! How value-driven strategies impact sentence memory. Memory & Cognition, 51(7), 1511–1526. 10.3758/s13421-023-01409-3, 37458967 PMC10915884

[bib13] Clewett, D., & Davachi, L. (2017). The ebb and flow of experience determines the temporal structure of memory. Current Opinion in Behavioral Sciences, 17, 186–193. 10.1016/j.cobeha.2017.08.013, 29276730 PMC5739077

[bib14] Clewett, D., DuBrow, S., & Davachi, L. (2019). Transcending time in the brain: How event memories are constructed from experience. Hippocampus, 29(3), 162–183. 10.1002/hipo.23074, 30734391 PMC6629464

[bib15] Clewett, D., Gasser, C., & Davachi, L. (2020). Pupil-linked arousal signals track the temporal organization of events in memory. Nature Communications, 11(1), 4007. 10.1038/s41467-020-17851-9, 32782282 PMC7421896

[bib16] Cohen, M. S., Rissman, J., Hovhannisyan, M., Castel, A. D., & Knowlton, B. J. (2017). Free recall test experience potentiates strategy-driven effects of value on memory. Journal of Experimental Psychology: Learning, Memory, and Cognition, 43(10), 1581–1601. 10.1037/xlm0000395, 28394160 PMC5624811

[bib17] Cohen, M. S., Rissman, J., Suthana, N. A., Castel, A. D., & Knowlton, B. J. (2014). Value-based modulation of memory encoding involves strategic engagement of fronto-temporal semantic processing regions. Cognitive, Affective, & Behavioral Neuroscience, 14(2), 578–592. 10.3758/s13415-014-0275-x, 24683066 PMC4074434

[bib18] Cowan, E. T., Chanales, A. J., Davachi, L., & Clewett, D. (2024). Goal shifts structure memories and prioritize event-defining information in memory. Journal of Cognitive Neuroscience, 36(11), 2415–2431. 10.1162/jocn_a_02220, 38991135

[bib19] Cowan, E. T., Schapiro, A. C., Dunsmoor, J. E., & Murty, V. P. (2021). Memory consolidation as an adaptive process. Psychonomic Bulletin & Review, 28(6), 1796–1810. 10.3758/s13423-021-01978-x, 34327677

[bib20] DuBrow, S., & Davachi, L. (2013). The influence of context boundaries on memory for the sequential order of events. Journal of Experimental Psychology: General, 142(4), 1277–1286. 10.1037/a0034024, 23957281 PMC3902141

[bib21] DuBrow, S., & Davachi, L. (2016). Temporal binding within and across events. Neurobiology of Learning and Memory, 134(Part A), 107–114. 10.1016/j.nlm.2016.07.011, 27422018 PMC5018468

[bib22] Dunsmoor, J. E., Murty, V. P., Clewett, D., Phelps, E. A., & Davachi, L. (2022). Tag and capture: How salient experiences target and rescue nearby events in memory. Trends in Cognitive Sciences, 26(9), 782–795. 10.1016/j.tics.2022.06.009, 35842373 PMC9378568

[bib23] Dunsmoor, J. E., Murty, V. P., Davachi, L., & Phelps, E. A. (2015). Emotional learning selectively and retroactively strengthens memories for related events. Nature, 520(7547), 345–348. 10.1038/nature14106, 25607357 PMC4432479

[bib24] Elliott, B. L., & Brewer, G. A. (2019). Divided attention selectively impairs value-directed encoding. Collabra: Psychology, 5(1), 4. 10.1525/collabra.156

[bib25] Ezzyat, Y., & Davachi, L. (2011). What constitutes an episode in episodic memory? Psychological Science, 22(2), 243–252. 10.1177/0956797610393742, 21178116 PMC4451827

[bib26] Foster, D. J., & Wilson, M. A. (2006). Reverse replay of behavioural sequences in hippocampal place cells during the awake state. Nature, 440(7084), 680–683. 10.1038/nature04587, 16474382

[bib27] Frank, D., Gray, O., & Montaldi, D. (2020). SOLID—Similar object and lure image database. Behavior Research Methods, 52(1), 151–161. 10.3758/s13428-019-01211-7, 30805861 PMC7005083

[bib28] Gershman, S. J., & Daw, N. D. (2017). Reinforcement learning and episodic memory in humans and animals: An integrative framework. Annual Review of Psychology, 68, 101–128. 10.1146/annurev-psych-122414-033625, 27618944 PMC5953519

[bib29] Gholston, A. S., Thurmann, K. E., & Chiew, K. S. (2023). Contributions of transient and sustained reward to memory formation. Psychological Research, 87(8), 2477–2498. 10.1007/s00426-023-01829-5, 37079090 PMC10116487

[bib30] Gomperts, S. N., Kloosterman, F., & Wilson, M. A. (2015). VTA neurons coordinate with the hippocampal reactivation of spatial experience. eLife, 4, e05360. 10.7554/eLife.05360, 26465113 PMC4695386

[bib31] Gruber, M. J., Ritchey, M., Wang, S.-F., Doss, M. K., & Ranganath, C. (2016). Post-learning hippocampal dynamics promote preferential retention of rewarding events. Neuron, 89(5), 1110–1120. 10.1016/j.neuron.2016.01.017, 26875624 PMC4777629

[bib32] Gruber, M. J., Watrous, A. J., Ekstrom, A. D., Ranganath, C., & Otten, L. J. (2013). Expected reward modulates encoding-related theta activity before an event. NeuroImage, 64, 68–74. 10.1016/j.neuroimage.2012.07.064, 22917987 PMC3518780

[bib33] Hayes, M. G., Kelly, A. J., & Smith, A. D. (2013). Working memory and the strategic control of attention in older and younger adults. Journals of Gerontology: Series B, 68(2), 176–183. 10.1093/geronb/gbs057, 22865825 PMC3693604

[bib34] Heusser, A. C., Ezzyat, Y., Shiff, I., & Davachi, L. (2018). Perceptual boundaries cause mnemonic trade-offs between local boundary processing and across-trial associative binding. Journal of Experimental Psychology: Learning, Memory, and Cognition, 44(7), 1075–1090. 10.1037/xlm0000503, 29461067 PMC6013306

[bib35] Igloi, K., Gaggioni, G., Sterpenich, V., & Schwartz, S. (2015). A nap to recap or how reward regulates hippocampal-prefrontal memory networks during daytime sleep in humans. eLife, 4, e07903. 10.7554/eLife.07903, 26473618 PMC4721959

[bib36] Jolly, E. (2018). Pymer4: Connecting R and Python for linear mixed modeling. Journal of Open Source Software, 3(31), 862. 10.21105/joss.00862

[bib37] Knowlton, B. J., & Castel, A. D. (2022). Memory and reward-based learning: A value-directed remembering perspective. Annual Review of Psychology, 73, 25–52. 10.1146/annurev-psych-032921-050951, 34587778

[bib38] Lee, A. K., & Wilson, M. A. (2002). Memory of sequential experience in the hippocampus during slow wave sleep. Neuron, 36(6), 1183–1194. 10.1016/S0896-6273(02)01096-6, 12495631

[bib39] Leonard, T. K., Mikkila, J. M., Eskandar, E. N., Gerrard, J. L., Kaping, D., Patel, S. R., Womelsdorf, T., & Hoffman, K. L. (2015). Sharp wave ripples during visual exploration in the primate hippocampus. Journal of Neuroscience, 35(44), 14771–14782. 10.1523/JNEUROSCI.0864-15.2015, 26538648 PMC4635128

[bib40] Lin, C. L., Wen, W., Cheng, P. X., Schallies, S., Grover, S., & Reinhart, R. M. G. (2025). Salient experiences enhance mundane memories through graded prioritization. Science Advances, 11(39), eady1704. 10.1126/sciadv.ady1704, 40991704 PMC12459409

[bib41] Lu, Q., Hasson, U., & Norman, K. A. (2022). A neural network model of when to retrieve and encode episodic memories. eLife, 11, e74445. 10.7554/eLife.74445, 35142289 PMC9000961

[bib42] McClay, M., Sachs, M. E., & Clewett, D. (2023). Dynamic emotional states shape the episodic structure of memory. Nature Communications, 14(1), 6533. 10.1038/s41467-023-42241-2, 37848429 PMC10582075

[bib43] Morse, S. J., Karagoz, A., & Reagh, Z. (2023). Event boundaries directionally influence item-level recognition memory. PsyArXiv. 10.31234/osf.io/h8bj2

[bib44] Murayama, K., & Kitagami, S. (2014). Consolidation power of extrinsic rewards: Reward cues enhance long-term memory for irrelevant past events. Journal of Experimental Psychology: General, 143(1), 15–20. 10.1037/a0031992, 23421444

[bib45] Murayama, K., & Kuhbandner, C. (2011). Money enhances memory consolidation—But only for boring material. Cognition, 119(1), 120–124. 10.1016/j.cognition.2011.01.001, 21292249

[bib46] Murphy, D. H., Schwartz, S. T., & Castel, A. D. (2024). Value-directed retrieval: The effects of divided attention at encoding and retrieval on memory selectivity and retrieval dynamics. Journal of Experimental Psychology: Learning, Memory, and Cognition, 50(1), 17–38. 10.1037/xlm0001264, 37326541

[bib47] Murty, V. P., & Adcock, R. A. (2014). Enriched encoding: Reward motivation organizes cortical networks for hippocampal detection of unexpected events. Cerebral Cortex, 24(8), 2160–2168. 10.1093/cercor/bht063, 23529005 PMC4089383

[bib48] Murty, V. P., LaBar, K. S., Hamilton, D. A., & Adcock, R. A. (2011). Is all motivation good for learning? Dissociable influences of approach and avoidance motivation in declarative memory. Learning & Memory, 18(11), 712–717. 10.1101/lm.023549.111, 22021253 PMC3207256

[bib49] Murty, V. P., Tompary, A., Adcock, R. A., & Davachi, L. (2017). Selectivity in postencoding connectivity with high-level visual cortex is associated with reward-motivated memory. Journal of Neuroscience, 37(3), 537–545. 10.1523/JNEUROSCI.4032-15.2016, 28100737 PMC5242406

[bib50] Nagel, J., Morgan, D. P., Gürsoy, N. Ç., Sander, S., Kern, S., & Feld, G. B. (2024). Memory for rewards guides retrieval. Communications Psychology, 2(1), 31. 10.1038/s44271-024-00074-9, 39242930 PMC11332070

[bib51] Nielson, K. A., & Bryant, T. (2005). The effects of non-contingent extrinsic and intrinsic rewards on memory consolidation. Neurobiology of Learning and Memory, 84(1), 42–48. 10.1016/j.nlm.2005.03.004, 15936682

[bib52] Patil, A., Murty, V. P., Dunsmoor, J. E., Phelps, E. A., & Davachi, L. (2016). Reward retroactively enhances memory consolidation for related items. Learning & Memory, 24(1), 65–69. 10.1101/lm.042978.116, 27980078 PMC5159660

[bib53] Poh, J.-H., Vu, M.-A. T., Stanek, J. K., Hsiung, A., Egner, T., & Adcock, R. A. (2022). Hippocampal convergence during anticipatory midbrain activation promotes subsequent memory formation. Nature Communications, 13(1), 6729. 10.1038/s41467-022-34459-3, 36344524 PMC9640528

[bib54] Shigemune, Y., Abe, N., Suzuki, M., Ueno, A., Mori, E., Tashiro, M., Itoh, M., & Fujii, T. (2010). Effects of emotion and reward motivation on neural correlates of episodic memory encoding: A PET study. Neuroscience Research, 67(1), 72–79. 10.1016/j.neures.2010.01.003, 20079775

[bib55] Shigemune, Y., Tsukiura, T., Kambara, T., & Kawashima, R. (2014). Remembering with gains and losses: Effects of monetary reward and punishment on successful encoding activation of source memories. Cerebral Cortex, 24(5), 1319–1331. 10.1093/cercor/bhs415, 23314939 PMC3977621

[bib56] Shigemune, Y., Tsukiura, T., Nouchi, R., Kambara, T., & Kawashima, R. (2017). Neural mechanisms underlying the reward-related enhancement of motivation when remembering episodic memories with high difficulty. Human Brain Mapping, 38(7), 3428–3443. 10.1002/hbm.23599, 28374960 PMC6866879

[bib57] Shohamy, D., & Adcock, R. A. (2010). Dopamine and adaptive memory. Trends in Cognitive Sciences, 14(10), 464–472. 10.1016/j.tics.2010.08.002, 20829095

[bib58] Silva, M., Baldassano, C., & Fuentemilla, L. (2019). Rapid memory reactivation at movie event boundaries promotes episodic encoding. Journal of Neuroscience, 39(43), 8538–8548. 10.1523/JNEUROSCI.0360-19.2019, 31519818 PMC6807272

[bib59] Silva, M., Wu, X., Sabio, M., Conde-Blanco, E., Roldan, P., Donaire, A., Carreño, M., Axmacher, N., Baldassano, C., & Fuentemilla, L. (2023). Neocortico-hippocampal ripple-based coordination during naturalistic encoding. bioRxiv. 10.1101/2023.09.27.559739

[bib60] Singer, A. C., & Frank, L. M. (2009). Rewarded outcomes enhance reactivation of experience in the hippocampus. Neuron, 64(6), 910–921. 10.1016/j.neuron.2009.11.016, 20064396 PMC2807414

[bib61] Sols, I., DuBrow, S., Davachi, L., & Fuentemilla, L. (2017). Event boundaries trigger rapid memory reinstatement of the prior events to promote their representation in long-term memory. Current Biology, 27(22), 3499–3504. 10.1016/j.cub.2017.09.057, 29129536 PMC6398599

[bib62] Spaniol, J., Schain, C., & Bowen, H. J. (2014). Reward-enhanced memory in younger and older adults. Journals of Gerontology: Series B, 69(5), 730–740. 10.1093/geronb/gbt044, 23690000

[bib63] Sterpenich, V., van Schie, M. K. M., Catsiyannis, M., Ramyead, A., Perrig, S., Yang, H.-D., Van De Ville, D., & Schwartz, S. (2021). Reward biases spontaneous neural reactivation during sleep. Nature Communications, 12(1), 4162. 10.1038/s41467-021-24357-5, 34230462 PMC8260738

[bib64] Swallow, K. M., Zacks, J. M., & Abrams, R. A. (2009). Event boundaries in perception affect memory encoding and updating. Journal of Experimental Psychology: General, 138(2), 236–257. 10.1037/a0015631, 19397382 PMC2819197

[bib65] van Dongen, E. V., Thielen, J.-W., Takashima, A., Barth, M., & Fernández, G. (2012). Sleep supports selective retention of associative memories based on relevance for future utilization. PLoS ONE, 7(8), e43426. 10.1371/journal.pone.0043426, 22916259 PMC3420871

[bib66] Villaseñor, J. J., Sklenar, A. M., Frankenstein, A. N., Levy, P. U., McCurdy, M. P., & Leshikar, E. D. (2021). Value-directed memory effects on item and context memory. Memory & Cognition, 49(6), 1082–1100. 10.3758/s13421-021-01153-6, 33638100

[bib67] Wang, Y. C., & Egner, T. (2022). Switching task sets creates event boundaries in memory. Cognition, 221, 104992. 10.1016/j.cognition.2021.104992, 34929522

[bib68] Wilhelm, I., Diekelmann, S., Molzow, I., Ayoub, A., Mölle, M., & Born, J. (2011). Sleep selectively enhances memory expected to be of future relevance. Journal of Neuroscience, 31(5), 1563–1569. 10.1523/JNEUROSCI.3575-10.2011, 21289163 PMC6623736

[bib69] Wittmann, B. C., Schott, B. H., Guderian, S., Frey, J. U., Heinze, H.-J., & Düzel, E. (2005). Reward-related fMRI activation of dopaminergic midbrain is associated with enhanced hippocampus-dependent long-term memory formation. Neuron, 45(3), 459–467. 10.1016/j.neuron.2005.01.010, 15694331

[bib70] Wolosin, S. M., Zeithamova, D., & Preston, A. R. (2012). Reward modulation of hippocampal subfield activation during successful associative encoding and retrieval. Journal of Cognitive Neuroscience, 24(7), 1532–1547. 10.1162/jocn_a_00237, 22524296 PMC3393089

[bib71] Yin, X., Havelka, J., & Allen, R. J. (2024). The role of attention and verbal rehearsal in remembering more valuable item-colour binding. Memory, 32(9), 1158–1172. 10.1080/09658211.2024.2389177, 39116079

