# Supplementary material for: Prioritizing Detailed Item Memories Through Post-Encoding Motivation Requires Consolidation
Source: Open Mind (Camb). 2026 Jul 17;10:1192–212. doi: 10.1162/OPMI.a.379 (PMC13518079; doi:10.1162/OPMI.a.379)
Supplement: Supplementary file 1 [file opmi-10-1192-s001.pdf]

Supplemental Information

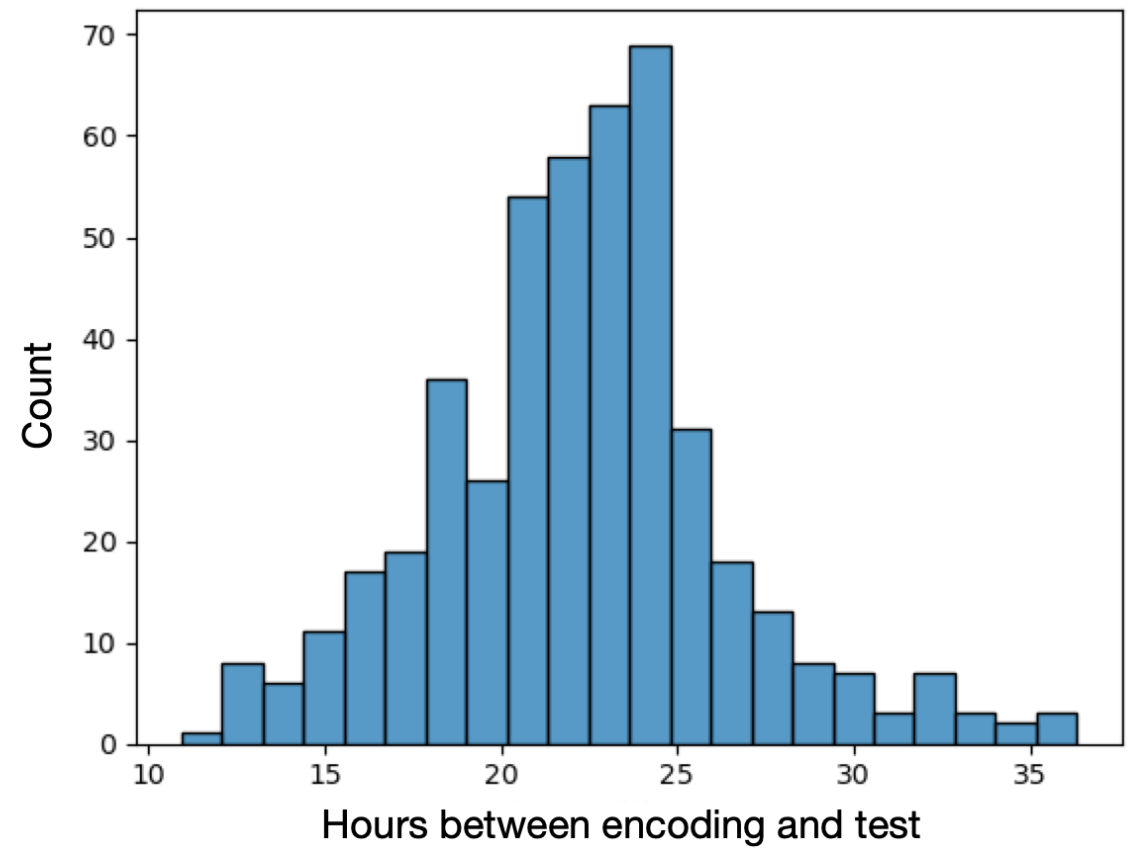

Supplementary Figure 1. *Hours between the encoding and test phases for participants in the delay condition.*

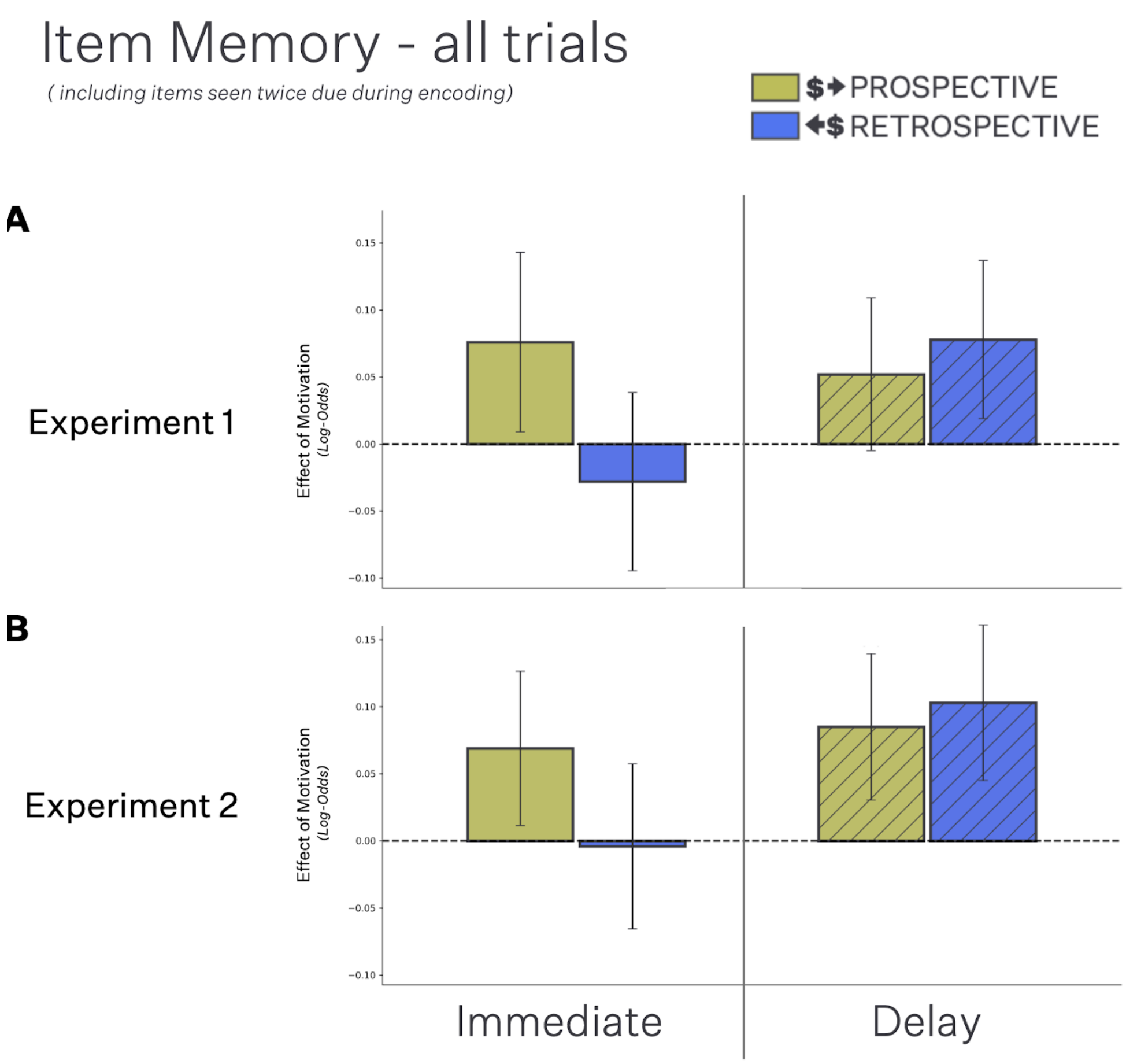

Supplementary Figure 2 Effect of motivation on item memory (all trials). *This figure shows the same results as in the main text, with the exception that the items that were seen twice during encoding*

(due to the temporal order task) are included here. Plotting conventions are the same as in the main text.

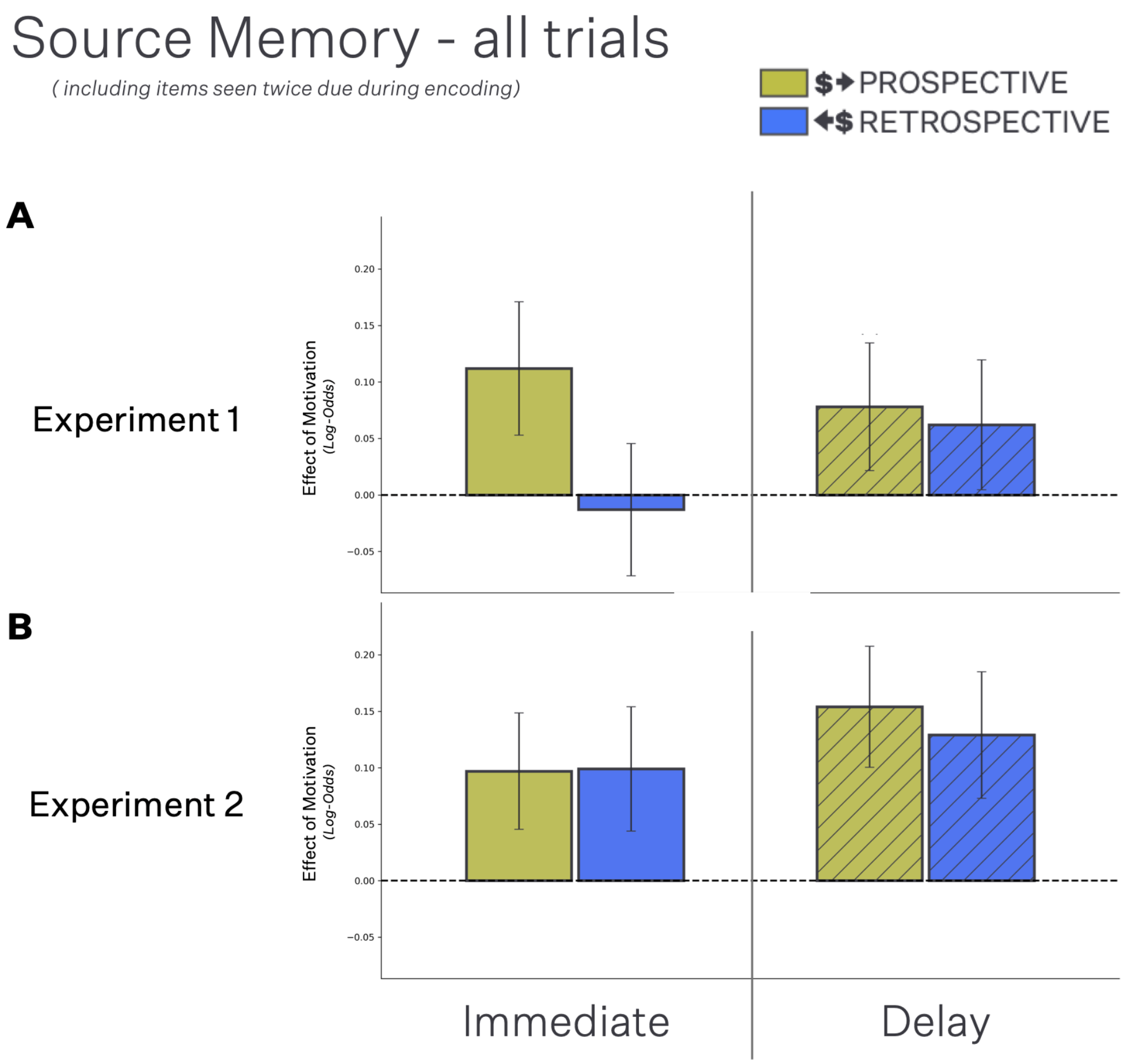

Supplementary Figure 3 Effect of motivation on source memory (all trials). *This figure shows the same results as in the main text, with the exception that the items that were seen twice during encoding (due to the temporal order task) are included here. Plotting conventions are the same as in the main text.*

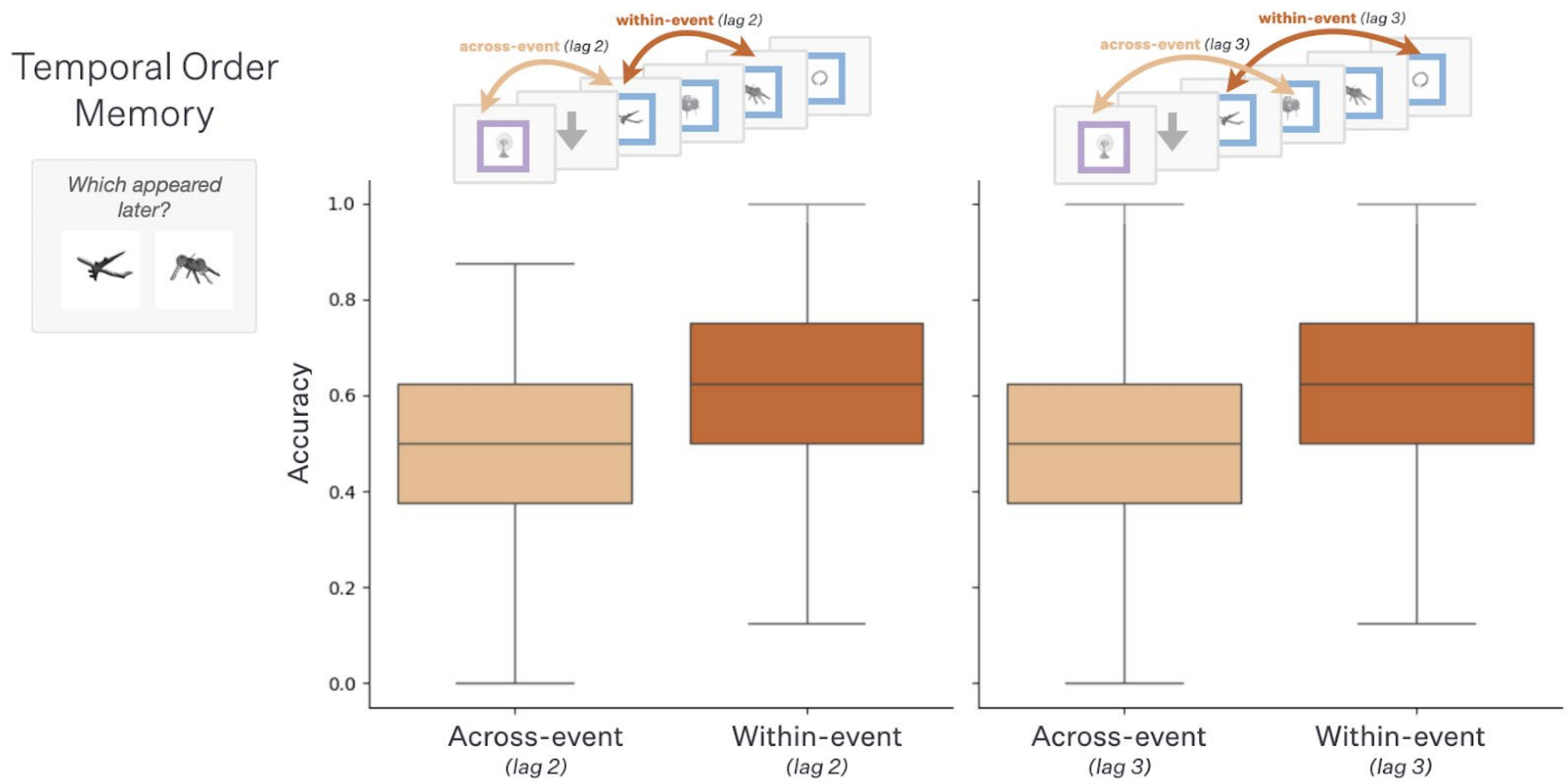

Supplementary Figure 4 Within and across-event temporal order memory. *Each boxplot represents the average accuracy for item pairs from within the same event and across two events. Our motivated encoding paradigm is designed to test how motivation affects episodic memory for items embedded in multi-stimuli events. In order to confirm this task induces event boundaries as intended, we wanted to replicate prior research demonstrating that temporal order memory is sensitive to event boundaries, such that memory for stimulus timing is enhanced for within-event judgements relative to across-event judgements (Clewett et al., 2019; Clewett & Davachi, 2017). That is, memory for the relative timing of two items is better when the items were from the same event rather than two separate events. As anticipated, the results of the temporal order test in Experiment 1 reveal significantly better memory for within-event comparisons relative to across-event comparisons. For both item pairs separated by one trial and those separated by two trials, memory accuracy is enhanced for within-event pairs (lag 2 - one trial in between: ($\beta = 0.62$, 95% CI [0.53, 0.72], $p < .001$); lag 3 - two trials in between:($\beta = 0.62$, 95% CI [0.53, 0.72], $p < .001$)*

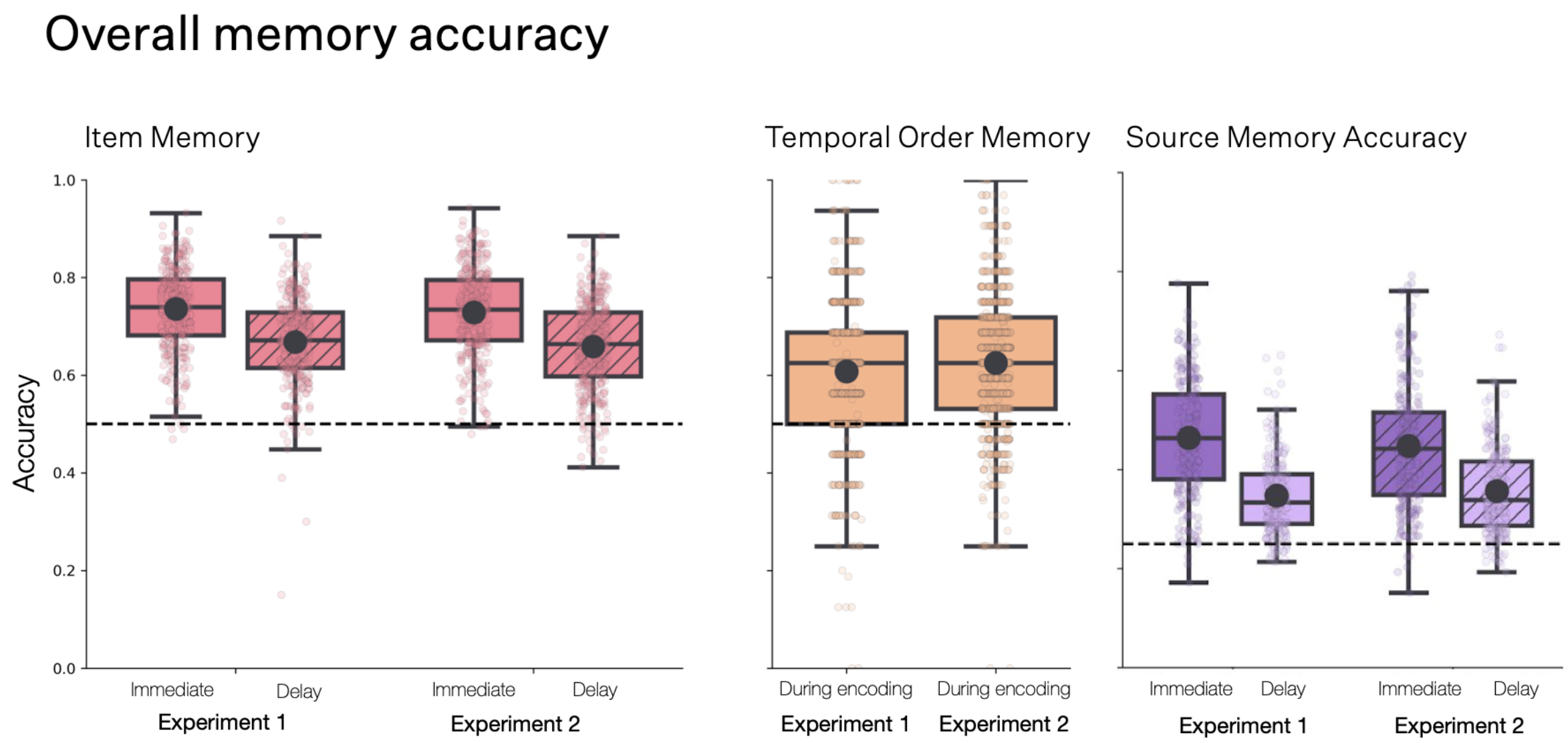

Supplementary Figure 5 Overall memory accuracy. *Memory accuracy overall for item (left) and temporal order (center) for experiments 1 and 2 and source memory (right). The dashed line reflects chance performance for each test.*

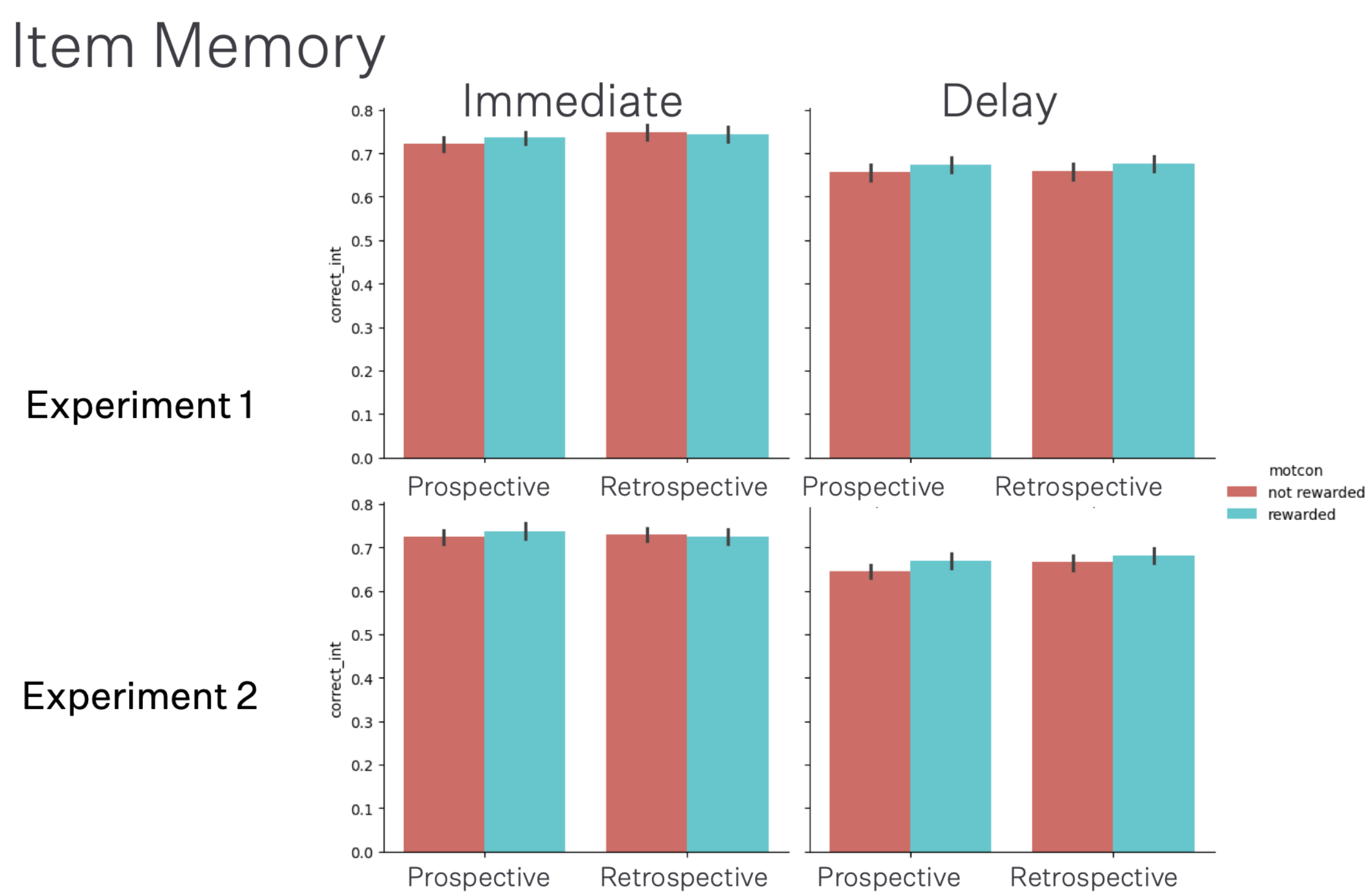

Supplementary Figure 6 Item memory accuracy by condition.

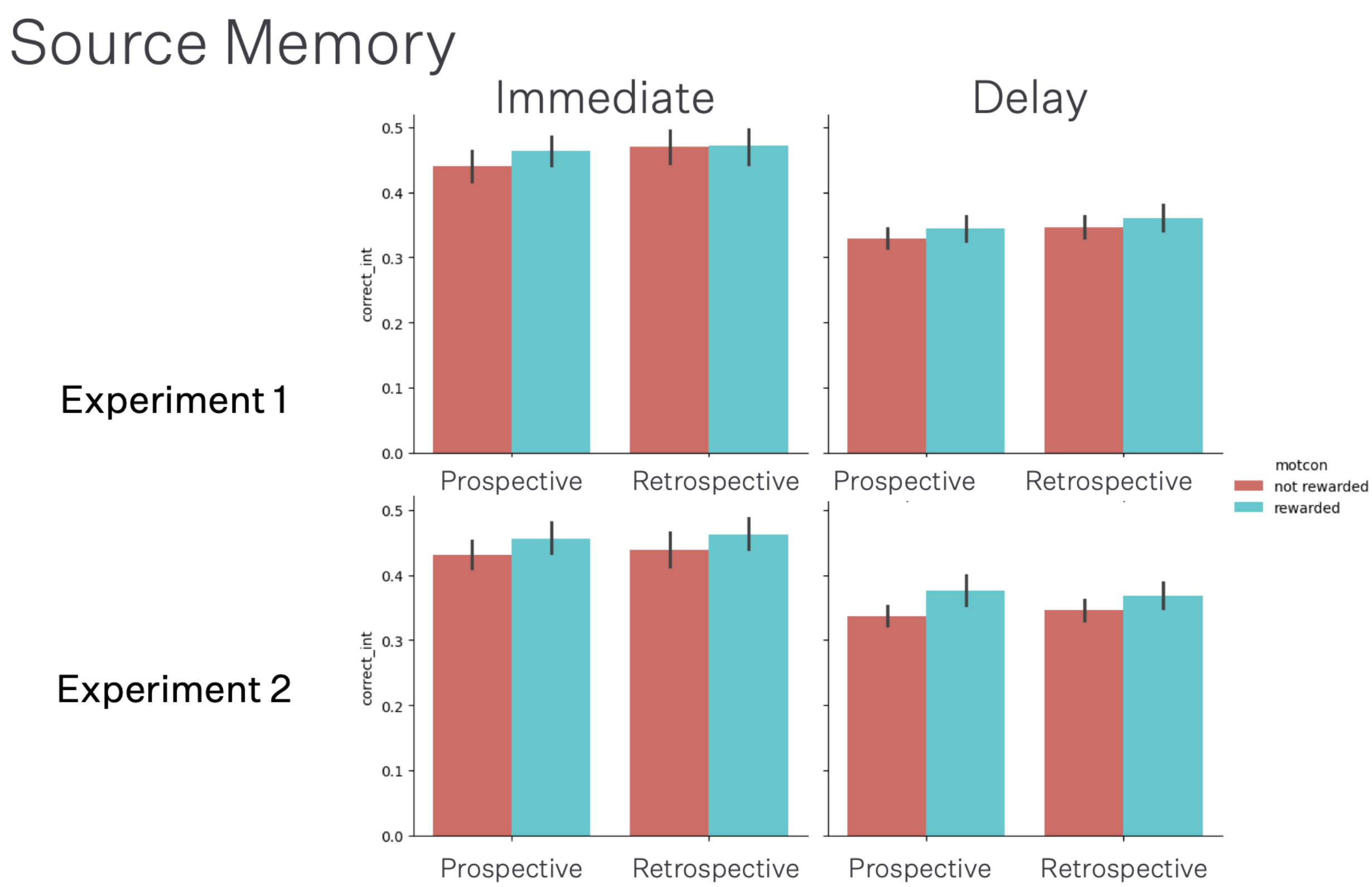

Supplementary Figure 7 Source memory accuracy by condition.

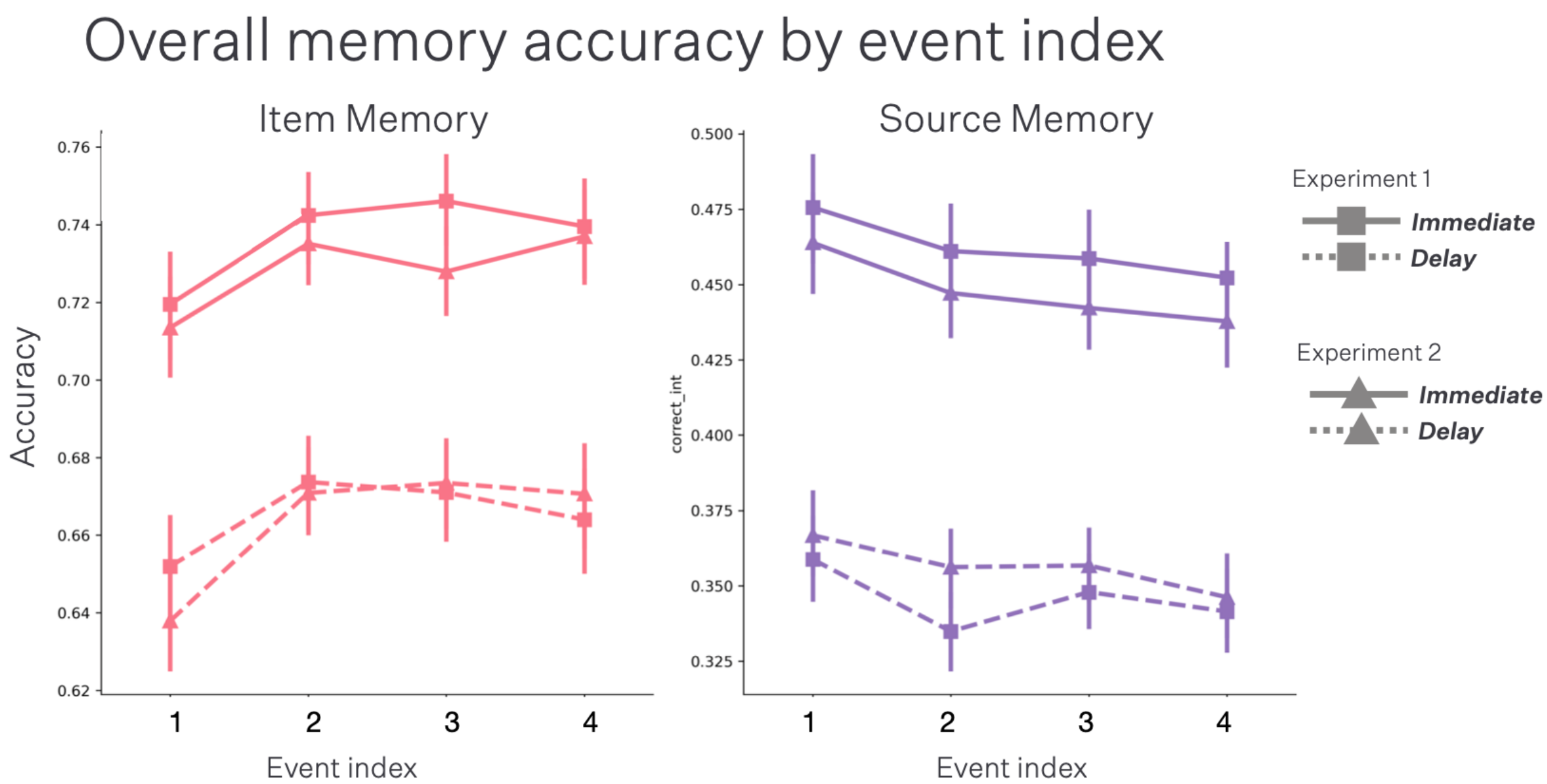

Supplementary Figure 8 Memory by position in event. *Each marker represents the average accuracy (across motivation conditions) for items at the given position in the event (1-4).*

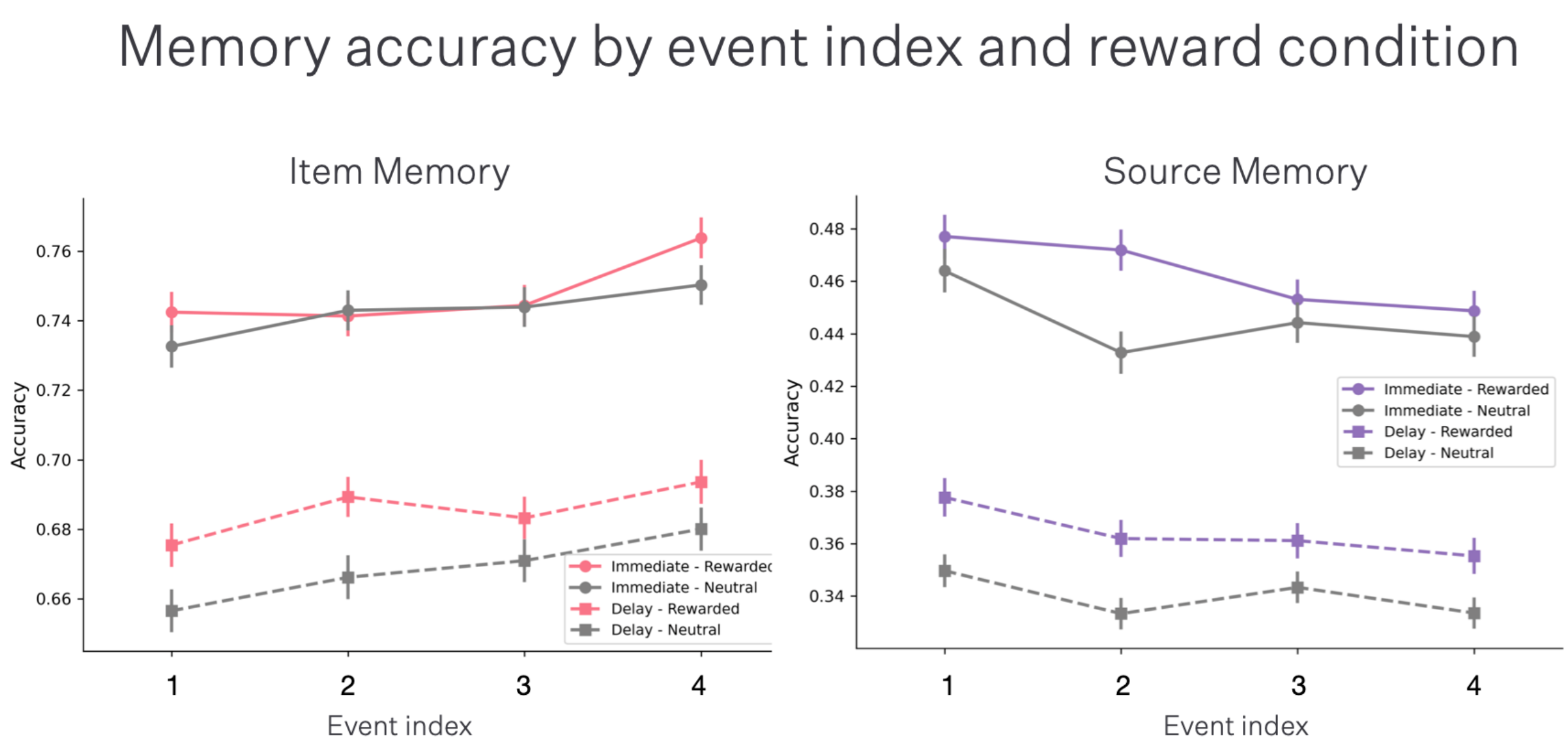

Supplementary Figure 9 *Each marker represents the average accuracy for items at the given position in the event (1-4). Gray lines are for items in neutral events, colored lines are for items in rewarded events.*

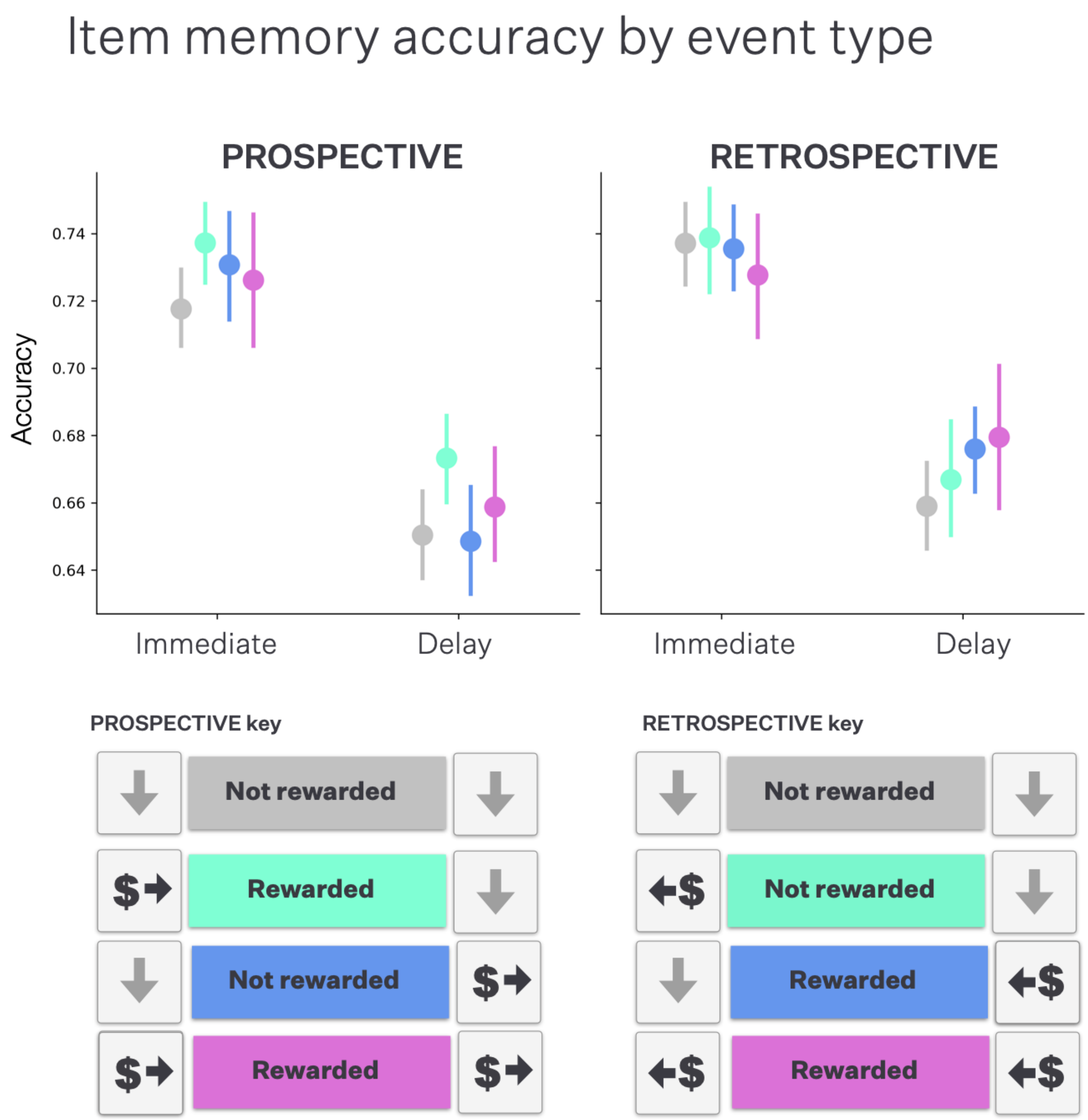

Supplementary Figure 10 Item memory accuracy by event type). Mean item memory accuracy split by event-type*:* 1. “reward-flanked” (reward cues on both sides), 2. “reward-before” (reward cue preceding the event), 3. “reward-after” (reward cue following the event) 4. “neutral flanked” (neutral cues on both sides).

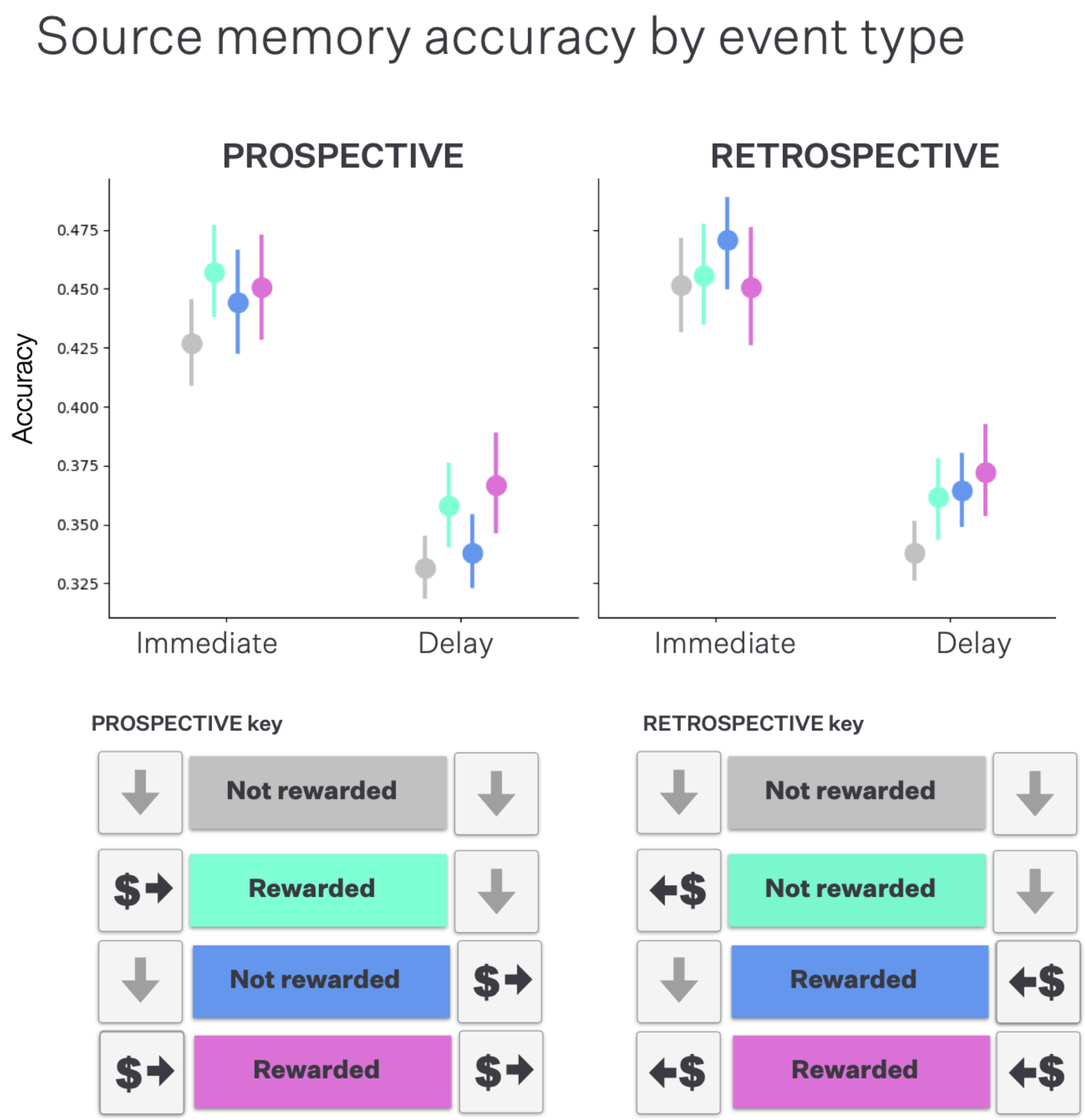

Supplementary Figure 11 Source memory accuracy by event type). Mean source memory accuracy split by event-type*:* 1. “reward-flanked” (reward cues on both sides), 2. “reward-before” (reward cue preceding the event), 3. “reward-after” (reward cue following the event) 4. “neutral flanked” (neutral cues on both sides).

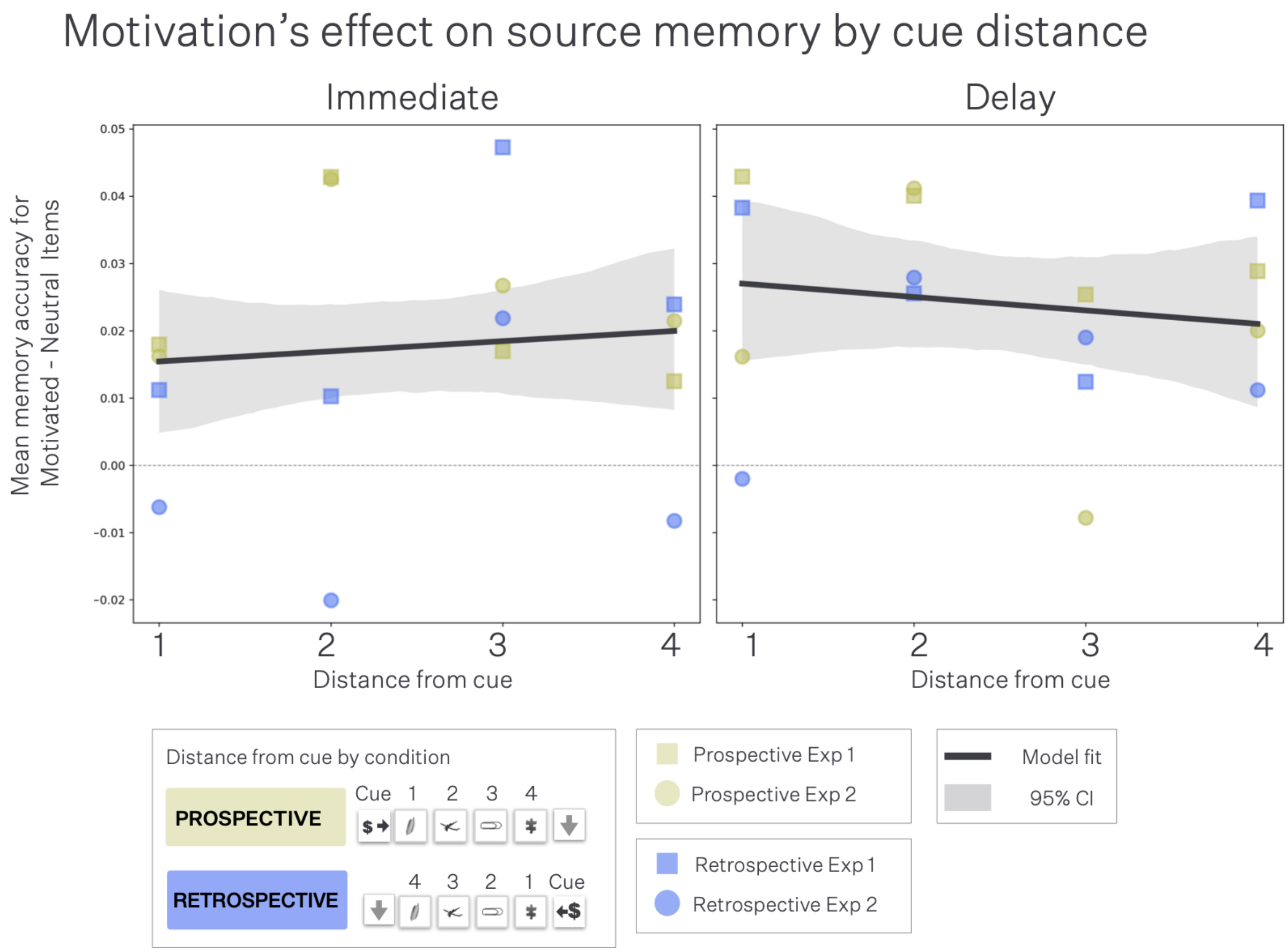

Supplementary Figure 12 Effect of motivation on source memory by distance from motivational cue. *Colored markers show the average difference in memory accuracy for motivated vs. neutral items, according to the item's distance from the cue (a distance of 1 is the item adjacent to the cue, a distance of 4 is the item farthest from the cue). Black lines show the model fit ('accuracy_difference ~ distance_from_cue') . Green and blue markers represent values for prospective and retrospective conditions, respectively; squares denote values for Experiment 1 while circles denote values for Experiment 2. We see no significant effect of distance in item memory measured immediately, but item memory measured after a delay shows a negative effect, such that the influence of motivation decreases with distance from the cue.*
